# Genome-wide analysis revealed the stepwise origin and functional diversification of HSDs from lower to higher plant species

**DOI:** 10.3389/fpls.2023.1159394

**Published:** 2023-06-15

**Authors:** Noor Saleem, Usman Aziz, Muhammad Ali, Xiangling Liu, Khairiah Mubarak Alwutayd, Rana M. Alshegaihi, Gniewko Niedbała, Amr Elkelish, Meng Zhang

**Affiliations:** ^1^ College of Agronomy, Northwest A & F University, Yangling, China; ^2^ College of Horticulture, Northwest A & F University, Yangling, China; ^3^ Department of Biology, College of Science, Princess Nourah bint Abdulrahman University, Riyadh, Saudi Arabia; ^4^ Department of Biology, College of Science, University of Jeddah, Jeddah, Saudi Arabia; ^5^ Department of Biosystems Engineering, Faculty of Environmental and Mechanical Engineering, Poznań University of Life Sciences, Poznań, Poland; ^6^ Biology Department, College of Science, Imam Mohammad ibn Saud Islamic University (IMSIU), Riyadh, Saudi Arabia; ^7^ Botany Department, Faculty of Science, Suez Canal University, Ismailia, Egypt

**Keywords:** HSDs, oil body, seed development, steroleosins, NADP(H), SDR

## Abstract

Hydroxysteroid dehydrogenase (HSDs) is an oil-body sterol protein (steroleosin) with an NADP(H) binding domain that belongs to the short-chain dehydrogenase/reductase (SDR) superfamily. There are numerous studies on the characterization of *HSDs* in plants. However, thus far, the evolutionary differentiation and divergence analysis of these genes remain to be explored. The current study used an integrated method to elucidate the sequential evolution of *HSDs* in 64 sequenced plant genomes. Analyses were conducted on their origins, distribution, duplication, evolutionary paths, domain functions, motif composition, properties, and cis-elements. Results indicate that except for algae, HSD1 was widely distributed in plant species ranging from lower to higher plants, while HSD5 was restricted to terrestrial plants, and HSD2 was identified in fewer monocots and several dicot plants. Phylogenetic analysis of HSD proteins revealed that monocotyledonous HSD1 in moss and ferns appeared closest to the outgroup, *V. carteri* HSD-like, *M. musculus* HSD1, and *H. sapiens* HSD1. These data support the hypothesis that *HSD1* originated in bryophytes and then in non-vascular and vascular plants, followed by *HSD5* only in land plants. Gene structure analysis suggests that *HSDs* in plant species came up with a fixed number of six exons, and the intron phase was primarily 0, 1, 0, 0, and 0. Similarly, duplication analysis revealed that segmental duplications were the main reason for *HSDs* in plant species. Physicochemical properties suggest that dicotyledonous HSD1s and HSD5s were mainly acidic. The monocotyledonous HSD1s and HSD2s and the dicotyledonous HSD2s, HSD3s, HSD4s, and HSD6s were mainly basic, implying that *HSDs* in plants may have a variety of functions. *Cis*-regulatory elements and expression analysis revealed that *HSDs* in plants might have roles in several abiotic stresses. Due to the high expression of *HSD1s* and *HSD5s* in seeds, these *HSDs* in plants may have roles in fatty acid accumulation and degradation.

## 1 Introduction

Triacylglycerols (TAGs) are the main components of the storage lipids packed into the organelles called lipid droplets (Miklaszewska et al., 2021). These droplets are covered by the phospholipid monolayer, which comprises embedded proteins. These proteins are termed oleosin, caleosin, and steroleosins (Chapman et al., 2012; Huang, 2018; Shao et al., 2019; Zienkiewicz and Zienkiewicz, 2020). The steroleosin protein is also known as sterol dehydrogenase, which is due to its sequence similarity with the hydroxysteroid dehydrogenase (HSD) family in mammals (Li et al., 2007; Shao et al., 2019). HSDs are related to the Aldo-Keto Reductase and short-chain dehydrogenase/reductase superfamilies (AKRs and SDRs) (D'andrea et al., 2007; Di Berardino et al., 2018; Bernardi et al., 2020). HSDs in the AKR superfamily have high affinity for NADP(H) and work in a reduction direction within cells (González-Thuillier et al., 2021). HSDs in the SDR superfamily are NAD(P)(H)-dependent oxidoreductases that can function either as ketosteroid reductases or as hydroxysteroid oxidases depending on whether they prefer NAD(P)(H) or NAD(H) (Penning et al., 2019; Ly et al., 2021).

In mammals, the HSD family is thought to have biological functions that involve modulating the steady-state concentrations of various steroid hormones through the interconversion of ketone and hydroxyl groups in the steroid’s backbone (Penning, 2003; Penning and Drury, 2007; Goodman, 2009; Henne et al., 2020). 17_estradiol is an estrogen-like hormone with a hydroxyl group at the 17th carbon position; in the presence of type 2 11β-HSD or type 4 17β-HSD, it can be dehydrogenated to form estrone, a less active ketone derivative (Penning, 2003; Penning and Drury, 2007; Goodman, 2009; Henne et al., 2020). Other *HSDs*, like 17-HSD1, can cause a reverse reaction to convert the ketone group on the estrone backbone into alcohol, thus regenerating the active hormone. Due to the high sequence similarity between the mammalian HSD and the steroleosin protein, it is generally believed that *in vitro* steroleosin in plants is hypothesized to have similar functions, such as the conversion of estradiol to the ketone estrone or cortisone (D'andrea et al., 2007; Shao et al., 2019).

Steroleosin has two domains: the sterol-binding dehydrogenase reductase domain and the N-terminal hydrophobic domain with the proline knot (Lin et al., 2002). To date, eight steroleosin genes are present in the Arabidopsis genome, and the role of steroleosin (*HSD1*) is also confirmed by the numerous studies in *Arabidopsis* plants (Lin et al., 2002; Lin and Tzen, 2004; Li et al., 2007; Shimada et al., 2008; Baud et al., 2009); i.e., there is a strong correlation between the fatty acids in seed oil bodies and the activation of *HSD1*. This is further supported by the fact that the *HSD1* gene is expressed in both the embryo and endosperm (Li et al., 2007; Baud et al., 2009). In addition, *AtHSD1* overexpression indicates that it influences germination and seed dormancy (Baud et al., 2009). These findings suggest that, like their mammalian counterparts, plant steroleosins may influence steroid signaling pathways by regulating the levels of biologically active hormones *via* chemical interconversions. Furthermore, the biological functions of *HSDs* are most likely involved in signal transductions regulated by their associated sterols (D'andrea et al., 2007). In mammals, plants, yeasts, and bacteria, the SDR metabolizes various substrates, such as steroids, monosaccharides, and flavonoids (Kallberg et al., 2002; Persson et al., 2009). Among these, steroids are catalyzed by *HSDs* and serve as important inter- and intracellular signal molecules in eukaryotes and prokaryotes. Some steroid hormones’ activation or deactivation may be regulated by prereceptors or intracrine regulatory mechanisms (Penning et al., 2000). For example, glucocorticoids, a steroid hormone that regulates cell proliferation and variation, are catalyzed at the prereceptor level by two isozymes of 11β-HSD. The type 1 isozyme is primarily found in tissues with a high level of glucocorticoid receptors, like the liver, adipose tissue, and gonads. It is mostly responsible for making cortisol, the active glucocorticoid. Even though Type 2 isozyme is overexpressed, it may work well as a barrier against cortisol at various concentrations (Blum and Maser, 2003). The differential regulation of these 11β-HSD isozymes is essential for cell proliferation and differentiation. Based on the discussion above, it was hypothesized that steroleosin-A and -B in sesame are associated with the activation of sterol signal transduction, which regulates specialized biological functions involved in the synthesis or mobilization of oil bodies during seed development or germination (Lin and Tzen, 2004).

The family Brassicaceae comprises many brassica crops as well as the model plant, *Arabidopsis thaliana*. Allotetraploid *B. napus* is the product of natural hybridization of the diploids *B. rapa* and *B. oleracea* approximately 7,500 years ago (Chalhoub et al., 2014; Shen et al., 2016). Approximately 12–20 million years ago (MYA), the segregation of Arabidopsis and Brassica plants occurred (Blanc et al., 2003; Town et al., 2006). Arabidopsis and its related subspecies, *Arabidopsis lyrata*, diverged 10 MYA (Hu et al., 2011). Brassica plants experienced a specific whole-genome triplication process from 5 to 15 MYA (Beilstein et al., 2010). The separation of *B. rapa* and *B. oleracea* occurred 4.6 MYA (Liu et al., 2014). The complete genomic sequence of the model plant provides an opportunity to map the evolution of the steroleosin gene family between *B. napus* and its progenitors. The genomic analysis identified as many as five steroleosins in *Brassica napus*, of which three have been verified by proteomic analysis. There is evidence for the roles of different steroleosins in model plants, but little information is available on the genomewide distribution of steroleosin in plant species. Questions like the presence of more steroleosin genes in Brassica species, their evolutionary processes, expression analysis, and biological functions still need to be answered. With the increased availability of genomic data, this work is now feasible. Thus, to better understand the origin of *HSD* genes in plant species, we performed phylogenetic analyses of the HSDs in plants, focusing mainly on previously underrepresented groups, such as algae, bryophytes, monilophytes, and “early-diverging” angiosperms. In addition, we analyzed the physiochemical properties, gene structure, and duplication events of *HSDs* in plant species. Together, these findings would be helpful in understanding the origin and functions of *HSDs* in plant species.

## 2 Research methodology

### 2.1 Identifying the HSD/HSD-like gene family in plants

To find *HSD* genes in plant species, the TAIR database was used to obtain the eight known Arabidopsis HSD protein sequences (http://arabidopsis.org/) (Huala et al., 2001). The retrieved sequences were subsequently used as queries in BLASTP searches of the Phytozome-12 database (https://phytozome.jgi.doe.gov/pz/portal.html/) (Goodstein et al., 2012). The analysis was carried out among all 64 sequenced plant species. The HSD protein sequences having E-values of less than 10^−10^ were further conformed within the species of plants. To further confirm the CDS of identified genes that were acquired from Phytozome-12, and in the TAIR database, these sequences were checked on BLASTX. The best-matched sequences with *At5g50600/At5g50700* (*HSD1*) (Jolivet et al., 2004; D'andrea et al., 2007), *At4g10020* (*HSD5*), *At5g50770* (*HSD6*), *At3g47350* (*HSD2*), *At3g47360* (*HSD3*), and *At5g50590/At5g50690* (*HSD4*) (Li et al., 2007; Baud et al., 2009) were selected for further research. To determine if the obtained sequences of HSDs contain SDR domains, all of the sequences were run through the smart-scan domain search tool (http://www.smart.embl-heidelberg.de/) (Finn et al., 2006), and sequences with the conserved domain PF00106 were selected. Similarly, the identified sequence of HSDs were also submitted to InterProScan (http://ebi.ac.uk/interpro) for the determination of the NADP(H) binding domain (IPR036291). *V. carteri* HSD-like was used as an outgroup, whereas due to the sequence similarity between plants and mammalian HSDs, *M. musculus* HSD1 (NCBI: Sequence ID NP_001038216.1) and *H. sapiens* HSD1 (NCBI: Sequence ID KAI2521299.1) were also included in the experiment, and their SDR and NADP(H) binding domains were also confirmed. There are variations in the nomenclature of *HSDs* in plant genomes. For this reason, we renamed the screened *HSD* and *HSD-like* genes according to their species names. The species was represented by the first letter of its scientific name and generic name. For instance, *Arabidopsis thaliana HSD1* and *Citrus clementina HSD1* are labeled as *A. thaliana HSD1* and *C. clementina HSD1*, respectively. The species contains multiple *HSD1* genes; English letters are appended to *HSD1* to differentiate it. For instance, *Musa acuminata* contains two *HSD1* genes, designated *M. acuminata HSD1a* and *M. acuminata HSD1b*. The remaining *HSDs* were also renamed in a similar way.

### 2.2 Construction of a phylogenetic tree and multiple sequence alignments

The NADP(H) binding domain is found in HSDs, which belong to the SDR superfamily (Lin et al., 2002; Yu et al., 2021). To construct phylogenetic trees, all the representative HSD sequences, along with *Volvox carteri* HSD-like, *M. musculus* HSD1, and *H. sapiens* HSD1, were uploaded to MEGA7 (Kumar et al., 2016), MUSCLE multiple sequence alignments were performed, and a neighbor-joining tree with a Poisson model was subsequently constructed. The value of bootstrap was adjusted to 1,000, whereas the rest of the parameters were left at their default settings. Using the iTOl-V5 online tool (https://itol.embl.de/), the phylogenetic tree was displayed and evaluated (Letunic and Bork, 2021).

### 2.3 Investigation of *HSD’s* gene duplication

It is expected that tandem and segmental duplications are key sources of gene expansion (Cannon et al., 2004). For the duplication studies, *HSDs* of various representatives were obtained as generic feature format version 3 (gff3) from the Phytozome 12 database, followed by analysis for the duplication. We selected plant genomes with chromosomal-level assembly and used MCScanX to examine the duplication of genes to confirm the validity of our findings (Wang et al., 2012). In plants, where the gene assembly’s level is low, the duplication events were not investigated.

### 2.4 Gene structure, conserved motifs, and *cis*-element analysis of HSDs

The gene structure display server (GSDS) at http://www.gsds.cbi.pku.edu.cn was used to determine the intron phase patterns (Hu et al., 2015). The HSD motifs were analyzed through MEME (http://meme-suite.org/tools/meme/) (Bailey et al., 2009). The following parameters were utilized: per sequence motif occurrence was either 0 or 1, 10 was the projected number of motifs, whereas the width of a motif ranged between 10 and 50 amino acids. InterPro (http://ebi.ac.uk/interpro/) was then used to annotate the identified motifs (Hunter et al., 2009). For *cis*-elements analysis of the *HSDs* and *HSD-like* genes, the upstream sequences (2 kb) were obtained from the phytozome 12 database, followed by submitting these sequences to the PromoterScan tool (https://www.bimas.cit.nih.gov/molbio/proscan/) and the Plant-Care tool (http://bioinformatics.psb.ugent.be/webtools/plantcare/html/) (Lescot et al., 2002).

### 2.5 Physiochemical properties and gene expression analysis of the *HSD*/*HSD-like*

To evaluate the physiochemical properties, the HSD proteins were submitted in ProtParam (an online tool: https://www.web.expasy.org/protparam/) to obtain the molecular weight (Mw), length, and theoretical isoelectric points (pIs) (Gasteiger et al., 2003). The expression patterns of HSDs were identified using an open-access transcriptome sequencing database. Since the *HSD*s are widely dispersed across plant species, the oil seed crop *Glycine max* was used as a reference species for analyzing expression patterns in various tissues. To explore non-oil expression patterns, *Amaranthus hypochondriacus* was used. Transcriptomic data were obtained from the JGIdatabase (https://www.genome.jgi.doe.gov/portal/).

## 3 Results

### 3.1 Identification of *HSD/HSD-like* from chlorophyta to angiosperm

**Figure 1:**
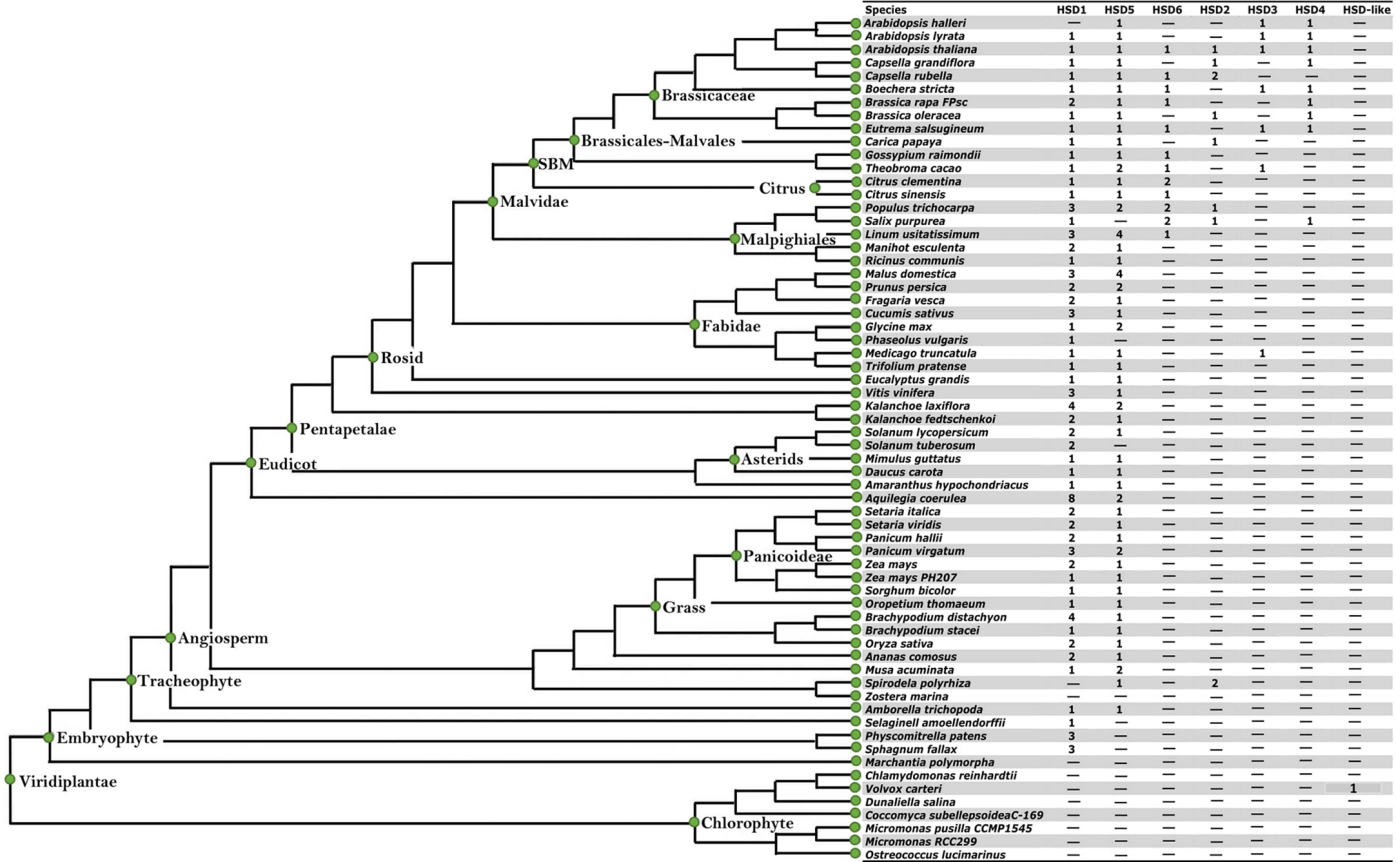
The identification of HSDs in plant species with sequenced genomes. The species tree is cited from the Phytozome (http://www.phytozome.net) and modified with full species names. The seven inserted columns (HSD1, HSD5, HSD6, HSD2, HSD3, HSD4 and HSD-like) were used for the identification of seed specific HSDs in plant species. The numbers within the column represent the presence and number of genes within the representative species. "-" represents that neither HSDs nor HSD-likes are present within the respective species.

To evaluate the structural characteristics and evolution of HSDs in plants, we analyzed all 64 plant species (11 chlorophytes, 3 bryophytes, 1 lycopodiophyte, 16 monocots, and 37 dicots) available in the Phytozome database. For the analysis, several factors were considered; for instance, do non-terrestrials have *HSD1, HSD2, HSD3, HSD4, HSD5*, and *HSD6* homologues? Are these genes present in all monocotyledonous and dicotyledonous plants? Which gene was the first to evolve in plants? Are the retrieved sequences suitable for further study? To date, eight HSD proteins have been identified in the TAIR genome (Lin et al., 2002; Li et al., 2007; Baud et al., 2009). Among the identified HSDs, two are homologous to AtHSD1 (At5g50600 and At5g50700) and AtHSD4 (At5g50590 and At5g50690) (Aziz et al., 2020). Therefore, in the current analysis, one protein sequence from each homologue was used to identify the respective HSDs (HSD1 and HSD4) in other plant species. The SDR domain (PF00106) and the NADP(H) binding domain (IPR036291) are the key domains of the HSD family. Thus, the existence of these domains was also confirmed by the analysis of retrieved sequences using the online tools Smart Domain Finder and InterProScan (Hunter et al., 2012). The summary of *HSDs* in plant species is presented in **Table 1**. The analysis revealed that *HSD* genes were absent from the algae. However, HSD proteins within the algae *V. carteri* genome were identified, and they carried NADP(H) and SDR domains (**Figure 1**; **Table S1A**). Two HSD1s were identified within two moss species (**Table S1A**), and HSD1s were widely distributed within terrestrial plants (**Table S1A**). HSD5 was restricted to land plants and, interestingly, in a relatively old gymnosperm, *Pine mannisona* (NCBI accession no. KT731102) (**Table S1A**). HSD2s were restricted to fewer monocots and dicots. HSD3s, HSD4s, and HSD6s were only restricted to a few dicots (**Tables S1A–F**). Sequence analysis revealed that some retrieved *HSD* sequences, for example, *A. comosus HSD5*, *C. grandiflora HSD5*, *A. halleri HSD5*, *M. truncatula HSD5*, and *T. pratense HSD5*, were missing either start or stop codons (**Table S1A**). Similarly, *B. rapa HSD6* and *B. rapa* FPSC *HSD3* lacked a stop codon (**Tables S1C, E**). For the sake of assurance, such sequences were deleted from later analysis. Similarly, HSD6 protein was identified in *Aquilegia coerulea*; however, when the coding sequence of *Aquilegia coerulea* was extracted and used for blast analysis on TAIR, HSD6 was identified as the fourth hit, whereas the best hit was HSD1, and such sequences were also not used in the analysis (**Tables S1A–F**). Collectively, our identification results suggest that among the HSDs in plant species, *HSD1* may have evolved first as it was distributed from relatively lower to higher plants, followed by *HSD5* and *HSD2*, respectively. The *HSD5* homologue was initially found in the gymnosperm *P. massoniana* but was later lost in other gymnosperms. *HSD6*, *HSD3*, and *HSD4* genes may have evolved solely in dicots.

**Table 1:** Summary of identified HSDs in plant species.

Group	Number of HSDs copies identified	Number of deleted HSDs sequence
Green algae *HSD-like*	1	0
Bryophyta *HSD1*	6	0
Monocotyledonous *HSD1s*	35	1
Dicotyledonous *HSD1*	69	0
Monocotyledonous *HSD2*	8	6
Dicotyledonous *HSD2*	14	6
Dicotyledonous *HSD3*	12	5
Dicotyledonous *HSD4*	16	7
Monocotyledonous *HSD5*	18	1
Dicotyledonous *HSD5*	50	5
Dicotyledonous *HSD6*	43	29

### 3.2 Phylogenetic analysis of HSDs in plant species

**Figure 2:**
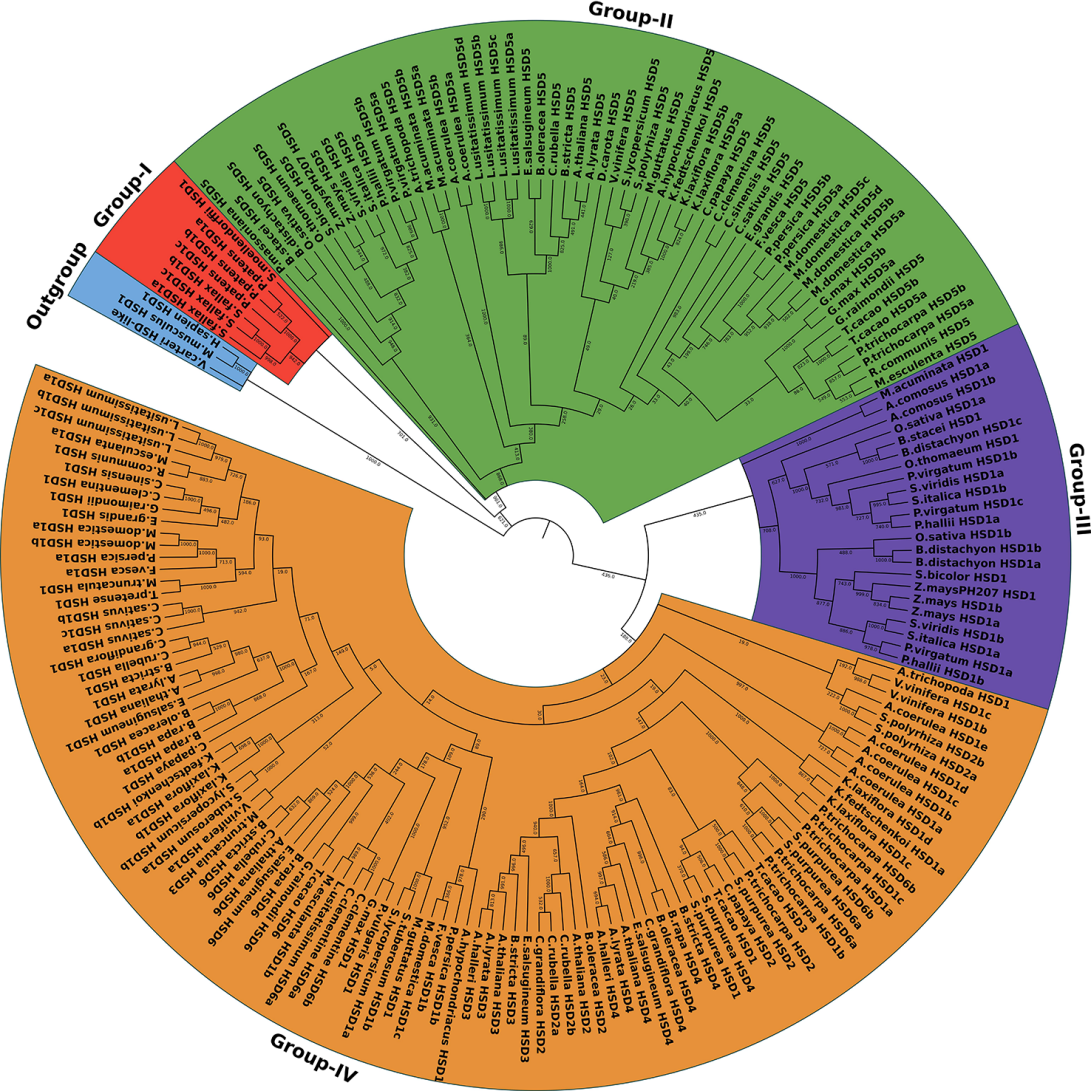
The phylogenetic relationship of the HSDs in plant species. The maximum likelihood tree was generated by MEGA. HSD-like in *Volvox carteri* along with *M. musculus* HSD1 and *H. sapien* HSD1 were used as an outgroup. The numbers on the branch represent the support values. The subfamilies are indicated by different colors and are numbered from I to IV.

To investigate the hypothesis of whether or not HSD1 evolved first in plants, all the identified HSDs, along with the outgroups, *V. carteri* HSD-like, *M. musculus* HSD1, and *H. sapiens* HSD1, were used for the phylogenetic analysis. As a result, the phylogenetic tree of the HSD family was categorized into four groups, numbers I–IV (**Figure 2**). The colors red, green, purple, and yellow represent clades I, II, III, and IV, respectively (**Figure 2**). HSD1s in *S. fallax*, *P. patens*, and *S. moellendorffii* of non-vascular land plants are clustered into clade I, which is closest to an outgroup. These data support the hypothesis that HSD1 originated in bryophytes and also confirms its presence in non-vascular land plants. Clade II comprises gymnosperm, monocotyledonous, and dicotyledonous HSD5 genes (**Figure 2**). They were closer to the *V. carteri* HSD-like, *M. musculus* HSD1, and *H. sapiens* HSD1, proposing that HSD5s were first evolved in gymnosperms, followed by monocots, and later appeared within dicots. Similarly, within clade III, monocotyledonous HSD1s are nearly grouped with HSD5s in terrestrial plants, suggesting that HSD1s in monocotyledonous plants are more closely related to HSD5s in land plants. Notably, *P. trichocarpa* HSD6a, b, and *S. purpurea* HSD6a, b, of the Malpighiales were found in clade IV, which suggests that HSD6s first appeared in this eudicot and later evolved in other dicots. *L. usitatissimum* HSD6a and b, *C. clementine* HSD6a and b, *C. sinensis* HSD6, *G. raimondii* HSD6, *T. cacao* HSD6, *B. rapa* HSD6, *E. salsugineum* HSD6, *A. thaliana* HSD6, *B. stricta* HSD6, and *C. rubella* HSD6 are located between the dicotyledonous HSD1s, demonstrating that HSD6 in dicots evolved simultaneously with HSD1s. Our results show similar results for the HSD3s and HSD4s (**Figure 2**). On the contrary, monocotyledonous *S. polyrhiza* HSD2 appeared in clade IV and is located between the monocotyledonous HSD1s, emphasizing that HSD2 first evolved within the monocots. Collectively, these results indicate that *HSD1* emerged first in plant species, followed by *HSD5, HSD2, HSD3, HSD4*, and *HSD6* (**Figure 2**).

### 3.3 Duplication events of *HSD genes* in plants

To comprehend the *HSD* gene family’s evolution adequately, we carried out duplication analyses on plants having multiple copies of *HSDs* by using MCScanX. As shown in **Table 2**, only *HSD1, HSD5, HSD2*, and *HSD6* were present within multiple species, and our results suggested that *HSD5, HSD6*, and *HSD2* were the results of segmental duplications, whereas *HSD1* was the outcome of segmental as well as tandem duplication events. The *HSD1s* in *B. stacei*, *P. hallii*, *S. viridis*, and *M. guttatus* were the results of tandem duplication events. On the contrary, segmental duplications were found in *A. coerulea HSD1*, *M. esculanta HSD1*, and *K. laxiflora HSD1*. The *HSD5s* in the dicotyledonous *P. trichocarpa* and *M. acuminata* were the results of segmental duplication events. Likewise, segmental duplications were the reason for *HSD6* in *P. trichocarpa* and *C. clementine*, and *HSD2* in *C. rubella* (**Table 2**).

**Table 2:** Gene duplication events of *HSD* gene family in plants.

Species	*HSDs*	Duplicated gene 1	Duplicated gene 2	Duplication event
*B. stacei*	*HSD1*	Brast09G060500.1	Brast09G060400.1	Tandem
*P. hallii*	*HSD1*	Pahal.G00866.1	Pahal.G00868.1	Tandem
*S. viridis*	*HSD1*	Sevir.7G082500.1	Sevir.7G082600.1	Tandem
*A. coerulea*	*HSD1*	Aqcoe5G348000.1	Aqcoe5G349000.1	segmental
*M. guttatus*	*HSD1*	Migut.I00555.1	Migut.I00556.1	Tandem
*M. esculanta*	*HSD1*	Manes.06G121600.1	Manes.06G121900.1	segmental
*K. laxiflora*	*HSD1*	Kalax.0535s0008.1	Kalax.0127s0013.1	segmental
*P. trichocarpa*	*HSD5*	Potri.019G073200.1	Potri.013G100200.1	segmental
*M. acuminata*	*HSD5*	Glyma.01G227900.1	Glyma.11G015100.1	segmental
*P. trichocarpa*	*HSD6*	Potri.015G099900.1	Potri.012G101900.1	segmental
*C. clementine*	*HSD6*	Ciclev10031927m	Ciclev10032335m	segmental
*C. rubella*	*HSD2*	Carubv10017691m	Carubv10017803m	segmental

### 3.4 Gene structure analysis of the *HSD/HSD-like*

**Figure 3:**
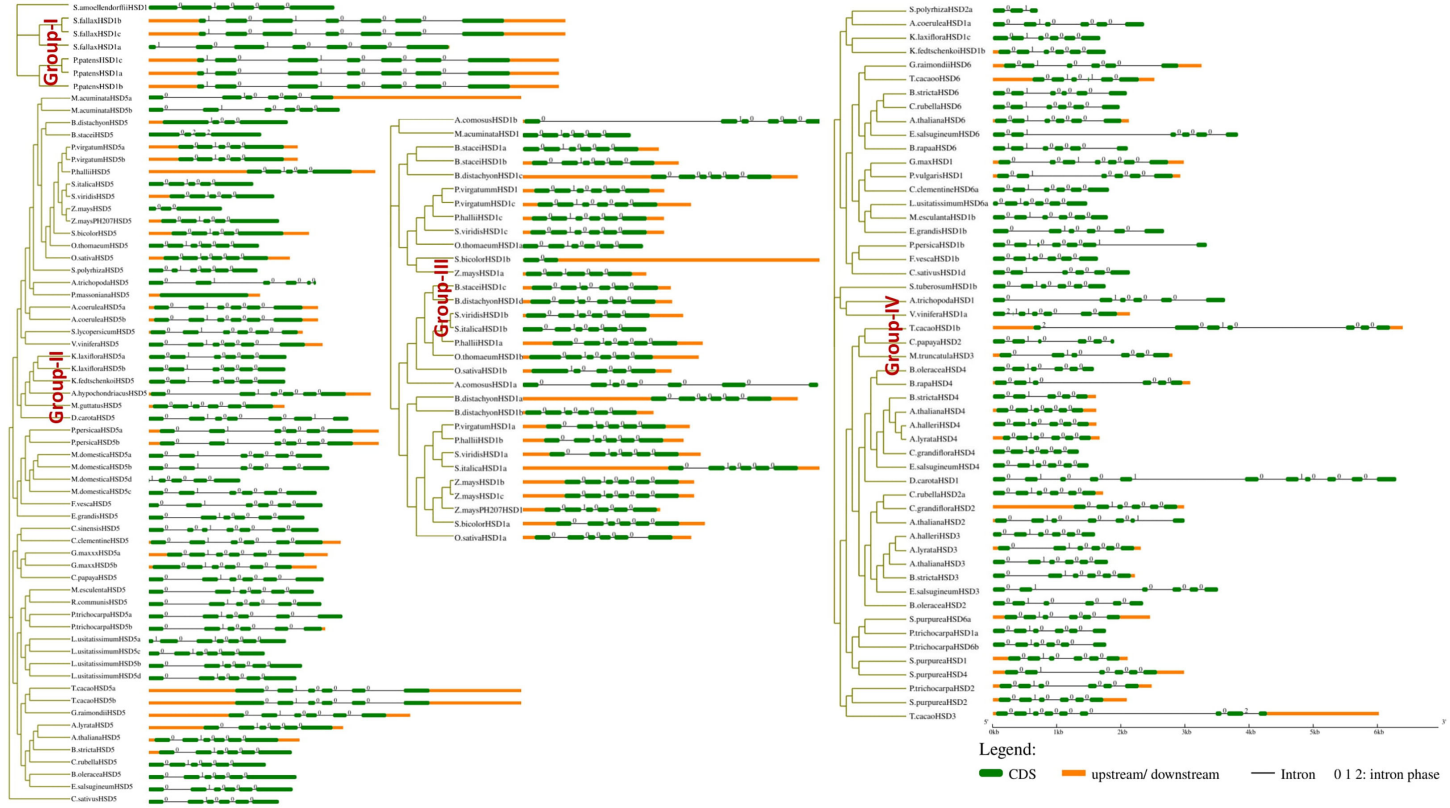
Intron phase of HSDs. Representative HSDs or HSD-likes are from subfamilies of the phylogenetic tree and was classified into four groups, numbers I to IV. The green block represents an exon, the black line represents an intron, and the yellow block represents an untranslated region. Phases of intron: 0 means intron between two consecutive codons, 1 means intron between the first and second nucleotide of a codon, and 2 means ‘intron’ between the second and third nucleotide of a codon.

For the determination of the conservation and structural variation of *HSD* gene families throughout the evolution cycle in plants, exon/intron phase diagrams were constructed, which thus assisted the phylogenetic classification of *HSD* and *HSD-like* genes. Subsequently, gene structure analysis was classified into four groups, numbers I to IV (**Figure 3**). Non-vascular land plants are clustered in Group I, and it was found that the *HSD1* in mosses, *S. fallax* and *P. patens*, came up with a set of seven exons, and the intron phase was 1, 0, 1, 0, 0, and 0 (**Figure 3**). Group II comprises gymnosperm and angiosperm *HSD5s*, and gene structure analysis reveals that, with some exceptions, as *HSD5* moved from monocots to dicots, it came up with a fixed number of exon and intron phases (0, 1, 0, 0, and 0). Moreover, it is worth noting that the first and last exons of *HSD5* in most of the higher plants were bigger than the rest. *A. trichopoda* is the earliest known basal angiosperm; the number of exons within *A. trichopoda HSD5* were 6, and the intron phase was made up of 0, 1, 0, 0, and 0 (**Figures 3**, **S1B, C**). With the development of *HSD5* in monocots *P. virgatum*, *P. hallii*, *S. italic*, *S. viridis*, *S. bicolor*, and *Z. mays PH 207*, the number of exons went down to five, with the last exon being bigger than the others. Similarly, as *HSD5* evolved within *B. stacie* and *B. distachyon*, the number of exons was further reduced to four with larger first and last exons, and the intron phase was 1, 0, and 0 (**Figures 3**, **S1B**). Among the dicots, *L. usitatissimum HSD5c* came up with seven exons, and the intron phase was 1, 0, 1, 0, 0, and 0. Within *M. domestica HSD5*, the number of exons was only four, and the intron phase was 1, 0, 0, and 0. *HSD5* has a fixed number of introns and exons compared to all other monocots and dicots. Taken together, these results suggest that with the evolution of *HSD5* from monocots to dicots, the intron and exon phases within plant species were conserved to carry out more diversified functions within their respective plant species.

Similarly, all the monocotyledonous *HSD1s* are located within group III (**Figure 3**). As *HSD1* evolved toward ferns and higher plants, one exon was lost, and the intron phase within most plant species was 0, 1, 0, 0, and 0 (**Figure 3**). During the transition from lower to higher plants, *HSD1s* underwent variations. For instance, within monocotyledonous *A. comosusHSD1a*, the number of exons was seven and the intron phase was 0, 1, 0, 0, 0, and 0 (**Figure 3**). Within dicots, the number of exons and intron phases among most species was fixed, but variations were observed. For example, within the eudicot *A. coerulea HSD1f* and *g*, the number of exons was eight and the intron phase was 2, 1, 0, 1, 0, 0 and 0. Similarly, the third intron phase of *A. coerulea HSD1f* and *g* was relatively larger, suggesting structural differences between the respective *HSD1s* (**Figure S1A**). Moreover, *V. vinifera HSD1c* and *T. cacao HSD1b* came up with seven exons, and the intron phase was 2, 0, 1, 0, 0, and 0. There were larger variations observed within a few dicotyledonous *HSD1s*; for example, *D. carota HSD1* and *A. hypochondriacus HSD1* had 11 and 13 exons, respectively, whereas the intron phase was 0, 1, 0, 0, 1, 0, 0, 0, and 0, 1, 0, 0, 1, 0, 0, 0, 2 (**Figure S1A**). Collectively, these results suggest that, with a few exceptions, especially within dicots, *HSD1s* from non-vascular plants to higher plants evolved with a fixed number of exons and introns.

Moreover, dicotyledonous *HSD2, HSD3, HSD4*, and *HSD6* appeared in group IV. The identification and phylogenetic results revealed that *HSD2, HSD3, HSD4*, and *HSD6* with differentiation evolved within the dicots only (**Figure 3**). The intron phase diagram suggests that all dicotyledonous *HSD6*s exhibit a fixed number of exon and intron phases except *L. usitatissimum HSD6*. The intron phase of *L. usitatissimum HSD6* was 0, 0, 0, 1, 0, 0, and 0 with eight exons (**Figures 3**, **S1C**). On the contrary, the intron phase of *HSD6* in all other dicots was 0, 1, 0, 0, and 0. The number of exons was restricted to six. It is worth noting that, when compared with other dicotyledonous *HSD6*, the second intron phase of *E. salsugineum HSD6* was relatively larger. Likewise, the second and fifth intron phases of *G. raimondii HSD6* were also larger, suggesting that during evolution, *HSD6s* within respective species have undergone structural differentiation. *HSD2* was identified in one monocot and several dicots (**Table S1D**). As shown in **Figure 3**, within the monocotyledonous *S. polyrhiza HSD2 a* and *b*, there were only three exons, and the intron phase was 0 and 1. During the evolution of *HSD2* from monocots to dicots*, HSD2* came up with a fixed number of six exons and introns, which were numbered 0, 1, 0, and 0. Notably, the third intron phase of *C. papaya HSD2*, *B. oleracea HSD2*, and *A. thaliana HSD2* (**Figure S1D**) were relatively larger than those of other dicots, indicating that the *HSD2* within dicots underwent structural differentiation during evolution. In the investigation of *HSD3s* and *HSD4s* conservation and structural variation in plant species’ evolutionary processes, an intron phase diagram was drawn from representative *HSD3s* and *HSD4s* from lower plants to higher plants. Both *HSD3s* and *HSD4s* were restricted to only a few dicots (**Figures 3**, **S1E, F**; **Tables SE, F**). Moreover, there are some variations in intron phase length among the *HSD3s* within plant species, but they came up with a fixed number of introns and exons; the intron phase of *HSD3s* in dicots was mainly 0, 1, 0, 0, and 0, and the number of exons was six (**Figure 3**). Similar to other *HSDs* within plant species, the intron phase of *HSD4s* within dicots was mainly 0, 1, 0, 0, and 0, and the number of exons was six. Moreover, the first, second, and last exons within most of the species were relatively larger (**Figure 3**). It is worth noting that the second intron phase of *S. purpurea HSD4* and the third intron phase of *B. oleracea HSD4* and *B. rapa HSD4* were relatively larger, suggesting that *HSD4s* within monocots underwent structural differentiation to carry out diverse functions within the respective plant species.

### 3.5 Motif analysis of HSDs during evolution

**Figure 4:**
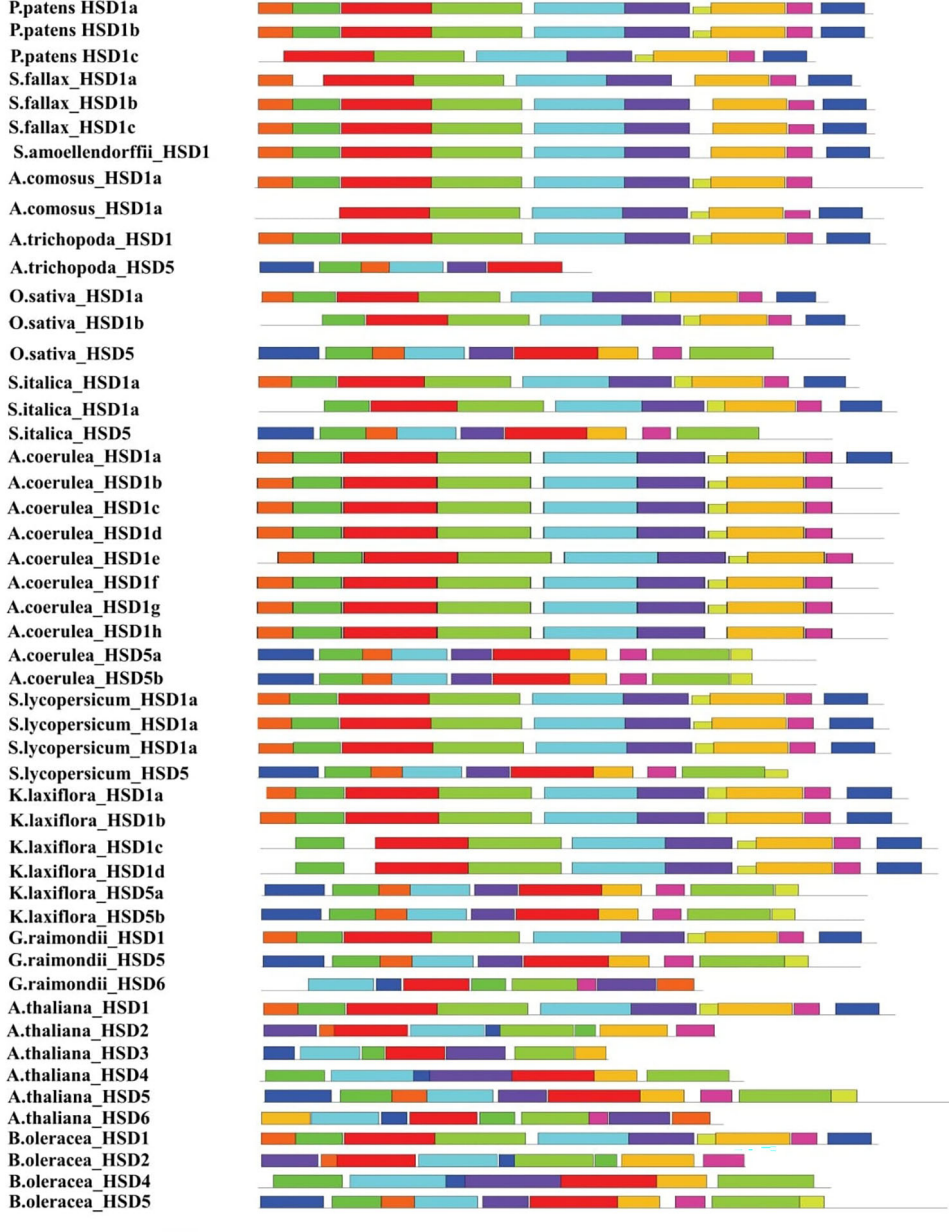
Motif patterns of representative HSDs/HSD-like from subfamilies of the phylogenetic tree. The conserved domains were identified using the MEME web server (http://meme-suite.org/tools/meme/). The ten identified motifs were represented in different colors.

To better understand the structural diversity of HSDs in plant species, the representative sequences of HSD1s were used for motif analysis through MEME (Bailey et al., 2009). Notably, outcomes revealed that among the 10 different identified motifs, motif 1, motif 2, and motif 3 (**Figures 4**, **S2A**), annotated as part of the SDR family (**Table 3**), were exclusively present among all the tested species. Motif 4 with unknown functions was present in all species except dicotyledonous *S. tuberosum* HSD1b. Similar to this, only one dicotyledonous *C. sinesis* HSD1 was lacking motif 5. Motif 6 was absent from two mosses, *P. patens* HSD1c and *S. fallax* HSD1a, and one monocot, *A. comosus* HSD1a (**Figures 4**, **S2A**). It was worth noting that motif 7 was absent from *A. comosus* HSD1a and from dicotyledonous *A. coerulea* HSD1a to h. This motif was part of lower vascular plants and several monocots, suggesting that, during evolution, motif 7 was lost from several dicots. Motif 8 was present among all species except the dicotyledonous *C. sinensis* HSD1. Moreover, motif 9 was absent from *P. patens* HSD1c of the embryophyte group and from several monocots and dicots, suggesting that this motif developed later during the evolution. Motif 10 was not found in *S. fallax* of Embryophyte, *S. moellendorffii* of Tracheophyte, and several dicots (**Figures 4**, **S2A**). This suggests that, during evolution, this motif developed in monocots, but as it evolved in dicots, it got lost in several dicotyledonous plants. From the 10 structural motifs in representative HSD5s, motif 1 (part of SDR) and motif 4, which has an unknown function, are found in all species. On the contrary, motif 2 was found only in *M. domestica* HSD5d, *C. sinesis* HSD5, and *Z. mays* HSD5, while motif 3, found only in *B. stacei* HSD5 and *A. trichopoda* HSD5, was found in all species. It is worth noting that motif 4 was found among all the verified plant species (**Figures 4**, **S2B**). The monocots *A. trichopoda* HSD5 and *B. stacei* HSD5 did not have motif 5, and neither did the dicot *V. vinifera* HSD5. This suggests that motif 5 developed later in monocots and, with little exception, evolved in dicots. Similar to this, motif 6 was absent from dicotyledonous *Z. mays* HSD5 and *M. domestica* HSD5d, suggesting that this motif also developed earlier within the monocots and later spread within the dicot plants. Motif 7, motif 8, and motif 9 were not part of the few monocotyledonous and dicotyledonous HSD5s, collectively suggesting that these motifs developed within the plant species, but during the evolution from monocots to dicots, these got lost from fewer species. It is worth noting that motif 10 was unique among the dicots only, whereas it was absent from monocots and the gymnosperm *P. massoniana* HSD5 (**Figures 4**, **S2B**), suggesting that during the evolution of HSD5 from monocots to dicots, this motif developed later only within the dicot plant species. Among HSD6s, the only species that did not have motif 4 were *C. sinesis* HSD6 and *C. clementine* HSD6. Similarly, motif 6 was lost within *E. salsugineum* HSD6 and *B. stricta* HSD6 only. Notably, motif 8, except for species from the Malpighiales group, was present among all species, and motif 10 was unique to this group only (**Figures 4**, **S2C**). Motifs 1 and 2, motif 4, and motif 9 were present among all the representative HSD2 species. Motif 3 and motif 6 were present within the dicotyledonous HSD2s only (**Figures 4**, **S2D**). Motif 7 was present among all the dicotyledonous HSD2s except *C. papaya* HSD2. Similarly, motif 5 and motif 8 were part of all identified dicotyledonous HSD2s except for *C. papaya* HSD2 and *C. rubella* HSD2b, respectively (**Figures 4**, **S2D**). It is important to note that motif 10 was only found in the monocotyledonous *S. polyrhiza* HSD2a and b. This suggests that this motif was lost in the dicotyledonous plants as HSD2 evolved from monocot to dicot. The motif analysis of HSD3s in the plant species is shown in **Figure 4** and **Supplementary Figure 2E**. The results showed that motif 2, motif 3, motif 4, and motif 6 were all present in all of the identified plant species. Motif 1 was among all the representative species except *M. truncatula* HSD3. Except for *T. cacao* HSD3 and *M. truncatula* HSD3, motif 7 was present in all the representative species. Interestingly, motif 9 and motif 10 were part of only *T. cacao* HSD3 and *M. truncatula* HSD3 (**Figures 4**, **S2E**), suggesting that, during evolution, these motifs got lost in the other dicotyledonous plants. **Figure 4** and **Supplementary Figure 2F** show that all of the representative HSD4s from different plant species have motifs 1, 2, 3, and 5. However, motif 4 was present among all the representative species except *B. stricta* HSD4. Similarly, all representative HSD4s were exhibiting motif 6 except *A. lyrata* HSD4 and *S. purpurea* HSD4. On the contrary, motif 7 was absent from *A. lyrata* HSD4 only (**Figures 4**, **S2F**). It is important to note that motif 8 was present only in *B. stricta* HSD4 and *S. purpurea* HSD4, whereas motif 9 and motif 10 were present only in *S. purpurea* HSD4. It is predicted that, during evolution, these motifs only evolved in dicotyledonous plants. Collectively, this discovery revealed that the SDR superfamily containing the NADP(H) binding domain is the same in all tested non-terrestrial and terrestrial plants.

**Table 3:** Annotation of identified HSDs motifs.

Motif no.	Width	Sequence	Annotation
1	35	GKVVLITGASSGIGEHLAYEYAKRGARLALVARRENSLREVADRARELGS	Short chain dehydrogenase
2	35	MDVNFWGSVYTTRFAJPHLKKSRGKIVVISSAASWLPAPRMSFYNASKAA	Short chain dehydrogenase
3	35	PDVJVIPADVSKPEDCKRFVDETINHFGRLDHLVNNAGIASVCMFEEIPD	Short chain dehydrogenase
4	35	LLNFFETLRVELGSDIGITIVTPGWIESEMTKGKFL	NA
5	26	RDAQVGPFPVESVEECAKAIVNSVCRGDRYLTEPAWFRATY	NA
6	35	LLLFLPPYYFFKLLLSILSSIFSEBV	NA
7	18	SETDALSKKJLDATGAKKVLYPSS	NA
8	22	WKVFCPEVLEWCYR	NA
9	16	MDLIHKFLNLVAPPFTFFS	NA
10	35	EGEMEVDQDM	NA

### 3.6 Physical and chemical properties of *HSDs* in plants

To figure out how they evolved, the physiochemical characteristics of HSDs in plants were further investigated for their physochemical properties (**Table 4**). Results showed that in bryophytes, the number of amino acids in HSD1s was between 309 and 342. In monocotyledonous and dicotyledonous plants, the number of HSD1s amino acids were between 347 and 406 and between 248 and 668, respectively. Moreover, the molecular weight of bryophytes ranged between 34.54 kDa and 38.3 kDa. Within monocotyledonous HSD1s, molecular length was between 38.35 kDa and 88.03 kDa, suggesting that there was variation between the molecular weights of HSD1s in monocots. Likewise, the molecular length of dicotyledonous HSD1s varied between 27.75 kDa and 73.82 kDa. The theoretical pI values of HSD1s in bryophytes, monocots, and dicots ranged from 8.64 to 9.14, 5.74 to 9.53, and 5.19 to 9.53, respectively. Monocotyledonous HSD1s are mainly basic in nature. However, several monocotyledonous HSD1s, i.e., *A. trichopoda* HSD1, *B. distachyon* HSD1a and c, *B. stacei* HSD1b, *O. sativa* HSD1a, *P. hallii* HSD1b, *P. virgatum* HSD1a, *S. italica* HSD1a, *S. viridis* HSD1a, *S. bicolor* HSD1a, *Z. mays* HSD1b, and c, and *Z. mays* PH207 HSD1a, were acidic. Similarly, among the 75 identified dicotyledonous HSD1s, the pI value of only 32 was greater than 7 (basic). Based on all of these results, it seems that HSD1s in lower plants and monocots are mostly basic, but as they move up into dicots, they become more acidic. The length of the proteins in monocotyledonous and dicotyledonous HSD5s varied a lot, from 223 to 451 residues for monocotyledonous HSD5s and from 311 to 412 residues for dicotyledonous HSD5. The molecular weight of monocotyledonous HSD5s was between 24.48 kDa and 48.7 kDa, whereas the dicotyledonous molecular weight ranged between 26.02 kDa and 51.98 kDa. The pI value of monocot HSD5 was between 6.11 and 7.25. Except for *M. acuminata* HSD5a and *D. distachyon* HSD5, all monocot HSD5s were acidic in nature. Similar to monocots, dicotyledonous HSD5s were also mainly acidic in nature, and the pI value ranged between 5.65 and 8.93 (**Table 4**).

**Table 4:** Physical and chemical properties of HSDs or HSD-like in plants.

Group	Protein length	Molecular mass	Theoretical pI	No. of Proteins with pI>7
Bryophyta *HSD1*	334.83±13	37769.24±1411.71	8.88 ±0.22	6
Monocotyledonous *HSD1s*	368.68±74.97	40763.23±8102.95.27	7.75±1.35	24
Dicotyledonous *HSD1*	350.59±43.04	39053.58±4718.3	7.13±1.33	32
Monocotyledonous *HSD5*	348.36±55.3	38333.93±5844.69	6.5±0.86	2
Dicotyledonous *HSD5*	364.13±31.44	40973.39±3501.6	6.58±0.81	9
Dicotyledonous *HSD6*	329.76±44.28	36685.63±4821.05	7.78±1.05	14
Monocotyledonous *HSD2*	164±0	18216.24±0	7.7±0	2
Dicotyledonous *HSD2*	297.5±22.76	33358.2888±2459.8	8.57±0.58	8
Dicotyledonous *HSD3*	325.43±48.43	36747.05286±5107.5	8.14±1.15	6
Dicotyledonous *HSD4*	294.56±14.73	32707.24±1599.55	9.01±0.18	9

HSD6s were only identified within dicot plants, and their protein length had between 311 and 480 amino acids, while their molecular weight was between 31.88 and 52.79 kDa. The pI value of HSD6s within dicots was between 5.28 and 9.08. Among all the identified HSD6s within dicots, the pI values of *C. sinensis* HSD6 and *E. salsugineum* HSD6 were 6.53 and 5.28, respectively, whereas the pI values of all others were greater than seven, suggesting that HSD6s within plant species are mainly basic in nature. Two copies of HSD2 were identified in monocotyledonous *S. polyrhiza* with a protein length of 164 and a molecular weight of 18.24 kDa. The pI value of *S. polyrhiza* HSD2a and b was 7.7, which means that HSD2 in the identified monocot was basic. Within dicots, the amino acid length of HSD2s ranged between 248 and 310, with a molecular weight of 24.85 kDa to 35.32 kDa. The pI value of HSD2s in dicotyledonous plants was between 7.67 and 9.64, which suggests that all HSD2s in plants are basic.

The amino acid length of dicotyledonous HSD3s ranged between 292 and 434, with a molecular weight of 33.12 kDa to 48.16 kDa. The pI values of HSD3s within dicots ranged between 6 and 9.77. It is worth noting that, except for *B. rapa* HSD3 (pI <6), all the identified HSD3s exhibit a higher than 7 pI value, indicating that HSD2s are mainly basic in nature. The amino acid length of dicotyledonous HSD4s ranged from 270 to 316, with molecular weights ranging from 30.92 kDa to 34.02 kDa. The pI values of HSD4s in dicots ranged from 8.72 to 9.1, which shows that most HSD4s are basic.

### 3.7 *Cis*-element regulation of *HSDs/HSD-like*

**Figure 5:**
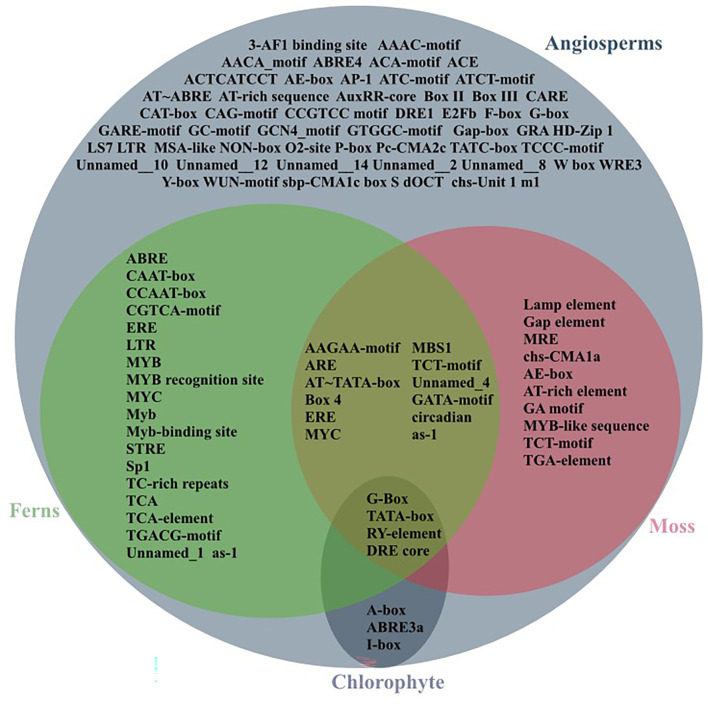
*Cis*-elements on the promoters of representative *HSDs/HSD-like* in some plants. The *cis*-elements were predicted by PLANTCARE. They were formed from Chlorophyte, moss, ferns and angiosperm. The cross section is the common *cis*-elements.

Analyses of *cis*-regulatory elements were done to explore the regulation and function of *HSDs* in plant species. The 2,000-bp upstream sequence of *HSDs* was obtained, and the PlantCARE tool was used to make predictions about *cis*-elements (**Table S2**). As shown in **Figure 5**, during the evolution of *HSDs* from lower to higher plants, the number of *cis*-elements gradually increased. From chlorophytes to angiosperms, the *cis*-elements light-responsive element (G-Box), the TATA-box, RY-element, and DRE core were found. However, the A-box, the ABA response element (ABRE3a), and the light-responsive element (I-box) were only found in chlorophytes and angiosperms, suggesting that during evolution, the *HSDs* in plants have undergone variations, which resulted in the loss of several *cis*-elements in moss and ferns (**Figure 5**). During evolution, moss, ferns, and angiosperms shared some unique *cis*-elements such as the light-responsive element (G-Box), TATA-box, the RY-element, and the DRE core, which were present in almost all plant species. On the contrary, the mosses, ferns, and angiosperms shared several of the same light-responsive elements (Box-4 and GATA motif), stress-related *cis*-elements (circadian, MBS1), and anaerobic induction (ARE) motifs. All of these results suggest that, as plants evolved and grew, the *cis*-regulatory elements in the *HSD* kept growing to serve a wider range of functions in different plant species. In moss and angiosperms, different light-responsive elements (AE-box, TCT motif, Lamp-element, and MRE), salicylic acid-responsive elements (TCA-element), and auxin-responsive elements (TGA) were found. With the evolution of *HSD cis*-elements in ferns, several other elements related to abiotic stress (ABRE, TCA-element, and TGACG motifs) were found. These results show that ferns’ *cis*-regulatory elements were mostly related to their ability to handle stress during evolution. Our analysis revealed several light-regulated and stress-related *cis*-elements that were unique to angiosperms only. Similarly, the *cis*–regulatory element related to endosperm expression (GCN4) was also found in the angiosperm *HSDs*, suggesting that *HSDs* in higher plants may have roles during seed development and for TAGs metabolism.

### 3.8 Expression analysis of the *HSD/HSD-like* gene

To see if the *HSD* genes’ projected cis-regulatory components are involved in chlorophyte growth, we retrieved and analyzed RNA-seq data from the JGI database of HSDs in *A. hypochondriacus* and *G. max* tissues (https://www.genome.jgi.doe.gov/portal/) (**Table S3**). Two HSDs, HSD1 and HSD5, are predominantly upregulated in seeds, which are the essential sites for TAG assembly and are considered to provide energy for developing seeds. Thus, the high expression level of two HSDs in seeds provides more evidence for their involvement in triacylglycerol metabolism. Fascinating to notice, *A. hypochondriacus* HSD1 and HSD5 are mainly upregulated in floral and seed tissues, while the maximum expression level of *G. max* HSD1 and HSD5a and b was only noticed in seed tissues (**Table S3**). This result is in line with the fact that several growth and development-related *cis*-elements have been found upstream of *HSD* genes. The roles of *HSD1* and *HSD5* genes in floral and seed tissues need intensive investigation.

## 4 Discussion

To date, eight HSD proteins based on SDR have been identified in the TAIR genome and HMM (Hidden Markov Model) (Lin et al., 2002; Li et al., 2007; Baud et al., 2009). *HSDs* are well studied in angiosperms as the primary regulators of oil accumulation. However, previous research has not examined the evolution of HSDs in plant species. Understanding the origin, evolution, and structural characteristics of HSDs is essential not only for their future functional evaluation in angiosperms but also for HSD exploring HSDs in non-vascular plants.

### 4.1 HSDs in plant species: Identification and evolution

This study found no HSDs in green algae, which suggests that HSDs may have developed later in plant species (**Figure 1** and **Table 1**). Surprisingly, some species exhibit uncertain sequences of HSD1; for instance, according to phytozome protein blast analysis, HSD1 was identified in *Z. marina.* However, when the CDS of *Z. marina* HSD1 were used for BLASTX (TAIR) analysis, this *HSD1* was identified as being 14 in number (**Table S1A**). From later analysis, such sequences were deleted and called “uncertain sequences”. Among the eight HSDs in Arabidopsis, only HSD1 was found in bryophytes and higher land plants. This suggests that HSD1 in plants may have been the first to evolve (**Figure 1** and **Table 1**). The HSD5s were limited to land plants (**Table S1B**) and in *P. massoniana*, which is a relatively old gymnosperm (Aziz et al., 2020). It is worth noting that no HSD was found in other gymnosperms, suggesting that HSD5 was only evolved in the pine genome and later lost in other gymnosperms. HSD2 was present in several dicots, and among monocots, it was present only in *S. polyrhiza* (**Table S1D**). Other HSDs, HSD6, HSD3, and HSD4, were restricted to fewer dicots only (**Tables S1C, E, F**). In a nutshell, identification results show that HSD1 was first evolved in lower and then in higher plants, followed by HSD5 and HSD2, respectively.

Previously, it was found that steroleosins in plants have homology with mouse and human HSDs. Thus, for phylogenetic analysis, *M. musculus* HSD1, *H. sapiens* HSD1, and *V. carteri* HSD-like were used as outgroups. Phylogenetic analysis of HSDs in plants also supported the identification results, as HSD1s from non-vascular land plants are clustered into clade I, which is closest to the outgroup (**Figure 2**). This finding supports the hypothesis that HSD1 first originated in bryophytes. Clade II comprises gymnosperm, monocotyledonous, and dicotyledonous plants. For instance, *P. massoniana* HSD5 and *A. trichopoda* HSD5 were closer to an outgroup than HSD1s from the lower plants, proposing that HSD5s were first evolved only in *P. massoniana*, followed by monocots, and later it appeared within dicots. Interestingly, *S. polyrhiza* HSD2 appeared between monocotyledonous HSD1 clades (**Figure 2**), suggesting that HSD2 first appeared in the monocots but has since disappeared within the other monocots. Malpighiales are one of the most diverse orders of angiosperms (Korotkova et al., 2009). Notably, *P. trichocarpa* HSD6a, b, and *S. purpurea* HSD6a, b, of Malpighiales appeared within clade IV, suggesting that HSD6s first appeared within this eudicot and later evolved within other dicots (**Figure 2**). In total, phylogenetic trajectory of all representative HSDs shows that HSD1 originated initially in the plant species, followed by HSD5, HSD2, HSD3s, HSD4s, and HSD6 (**Figure 2**).

The evolutionary changes in the gene family can also be assessed by intron and exon modifications, such as deletion or insertion of introns and exons (Xu et al., 2012). The above results were further supported by gene structure analysis, where the monocotyledonous *S. italica HSD5* has five exons and the intron phase is 0, 1, 0, and 0. To differentiate dicots from monocots, during the evolution of *HSD5*, the number of exons increased to six, and the intron phase was mainly 0, 1, 0, 0, and 0 (**Figure 3**). It is intriguing to notice that *HSDs* in vascular plants have evolved with some characteristics in common throughout evolution. Similarly, the intron phase of *HSD1s* in bryophytes was 1, 0, 1, 0, 0, and 0, with little variation as *HSD1s* shifted from lower to higher plants. All of the known *HSDs* had mostly 0, 1, 0, 0, 0, and 0 as their intron phase. Furthermore, with a few exceptions, all *HSDs* within plant species have six exons, indicating that steroleosins play similar roles in the plant species (**Figure 3**).

### 4.2 Potential roles of HSDs in plant species

Among the 10 different motifs identified within representative species, motif 1, motif 2, and motif 3 were annotated as being part of the SDR family (**Table 3**) and were present among all the tested species. However, the function of the remaining motifs is unknown (**Figure 4**; **Table 3**). Our findings revealed that the SDR superfamily exhibiting the NADP(H) binding domain remains the same among all the tested non-terrestrial and terrestrial plants. Interestingly, with the evolution of HSD5s from monocots to dicots, the number of motifs continuously increased (**Figure 4**), showing their diverse function in plants. DNA segments that are nearly identical (90%–100%) and found on multiple genomic sites are referred to as segmental duplications (Khaja et al., 2006). Previous studies suggest that within Arabidopsis, AtHSD1a and AtHSD4a are found to be segments of AtHSD1b and AtHSD4b, respectively, which is due to the 33-kb duplication events on chromosome 5 (Baud et al., 2009; Aziz et al., 2020). Our study revealed that both segmental and tandem duplication were the reasons for HSD1s in plants. However, HSD2s, HSD5s, and HSD6s were the result of segmental duplication (**Table 2**). According to physiochemical studies, except for HSD5, all the other HSDs were mainly basic in nature (**Table 4**). These results collectively suggest that steroleosins may have diversified roles within plant species (Aziz et al., 2020). According to the upstream sequence analyses, the majority of the HSDs within different plant species have a *cis-*element such as light-responsive element (G-Box), a TATA-box, an RY-element, and a DRE core. However, A-box, the ABA response element (ABRE3a), and the light-responsive element (I-box) were only found in chlorophytes and angiosperms, suggesting that, during evolution, the *HSDs* in plants have undergone variations that resulted in the loss of several *cis*-elements in moss and ferns (**Figure 5**). On the contrary, the mosses, ferns, and angiosperms shared several of the same light-responsive elements (Box-4 and GATA-motif), stress-related *cis*-elements (circadian, MBS1), and anaerobic induction (ARE) motifs. Previously, it has been observed that GATA participates in anaerobic environments (Ahmad et al., 2019). For the response to light signals, the GATA element collaborates with the G-box or GT1 motif (Luo et al., 2010; Liu et al., 2019). These outcomes collectively indicate that during evolution and as per growth requirements, the HSD’s *cis*-regulatory elements continued to increase for various functions. It is interesting to note that with the evolution of *HSD cis*-elements in ferns, several other abiotic stress-related elements (ABRE, TCA-element, and TGACG motifs) were also identified. These results suggest that during evolution, the *cis*-regulatory elements in ferns were mainly related to stress tolerance.

Expression analysis of two *HSD* genes, *HSD1* and *HSD5*, shows that they are predominantly upregulated in seeds, which are the essential site for TAG assembly and are considered to provide energy for developing seeds. Thus, the high expression level of two *HSDs* in seeds provides more evidence for their involvement in triacylglycerol metabolism (Aziz et al., 2020). Fascinating to notice, *A. hypochondriacus HSD1* and *HSD5* are mainly upregulated in floral and seed tissues, while the maximum expression level of *G. max HSD1* and *HSD5*a and b was only noticed in seed tissues (**Table S3**). This result is consistent with several *cis*-elements relevant to growth and development being identified upstream of *HSD* genes. In the future, the roles of *HSD1* and *HSD5* genes in floral and seed tissues will need intensive investigation.

## 5 Conclusion

Genome-wide identification and phylogenetic tree analysis revealed that among several HSDs in plants, HSD1s were first evolved from lower to higher plants, followed by HSD5s in terrestrial plants only. Similarly, with little distinction, HSD2s, HSD3s, HSD4s, and HSD6s evolved later in plant species. Gene structure analysis suggested that, during evolution, HSDs in plants came up with a fixed number of exons (6) and the intron phase was primarily 0, 1, 0, 0, and 0. Among all the HSDs in plants, HSD1s within dicots and HSD5s within land plants were mainly acidic in nature. However, the monocotyledonous HSD1s and HSD2s and the dicotyledonous HSD2s, HSD3s, HSD4s, and HSD6s were mainly basic in nature, suggesting that HSDs in plants may have diversified roles. *HSDs-cis-elements* and expression analysis revealed that *HSD1* and *HSD5* may have roles in the accumulation or degradation of fatty acids in seeds, which need to be further elucidated.

## Data availability statement

The raw data supporting the conclusions of this article will be made available by the authors, without undue reservation.

## Author contributions

NS: conceptualization, data curation, formal analysis, investigation, methodology, software, writing—original draft preparation, and writing—review and editing. UA: validation, investigation, and writing—review and editing. MA: conceptualization, writing, and editing. XL: data curation and validation. KA: validation and investigation. RA: data curation. GN: investigation. AE: data curation and formal analysis. MZ: conceptualization, funding acquisition, investigation, methodology, project administration, and supervision. All authors contributed to the article and approved the submitted version.
