# Supplementary material for: Genome-wide analysis revealed the stepwise origin and functional diversification of HSDs from lower to higher plant species
Source: Front Plant Sci. 2023 Jun 15;14:1159394. doi: 10.3389/fpls.2023.1159394 (PMC10311447; doi:10.3389/fpls.2023.1159394)
Supplement: Supplementary file 1 [file DataSheet_1.doc]

Supplementary Material

**Genome-wide analysis revealed stepwise origin and functional diversification of HSDs from lower to higher plant species**

**Noor Saleem^1†^, Usman Aziz^1†^, Muhammad Ali^2^,** **Xiang ling Liu^1^, Khairiah Mubarak Alwutayd^3^**

**, Rana M. Alshegaihi^4^, Gniewko Niedbała^5^, Amr Elkelish^6,7^, Meng Zhang^1^***

*** Correspondence:** Corresponding Author: [zhangm@nwsuaf.edu.cn](mailto:zhangm@nwsuaf.edu.cn)

# Supplementary Figures and Table

## Supplementary Figures


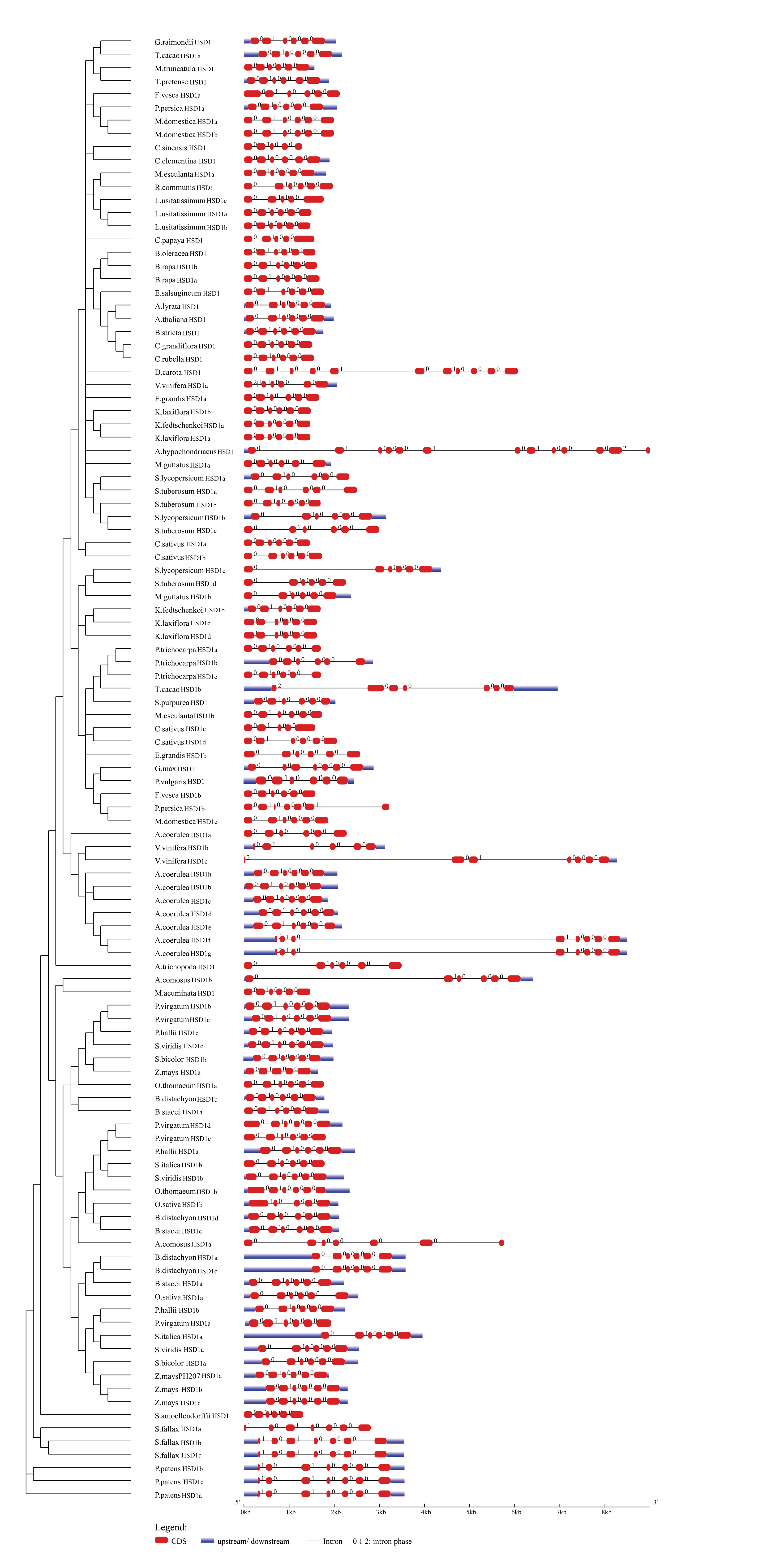


**A**

**Supplementary Figure 1.** Intron phase analysis of HSDs. **(A)** Gene structures of *HSD1s*. The red blocks represent exon, the black lines represent intron and blue blocks represent untranslated regions. Phases of introns: **0** means introns between two consecutive codons, **1** means intron between first and second nucleotide of a codon and **2** means intron between second and third nucleotide of a codon.


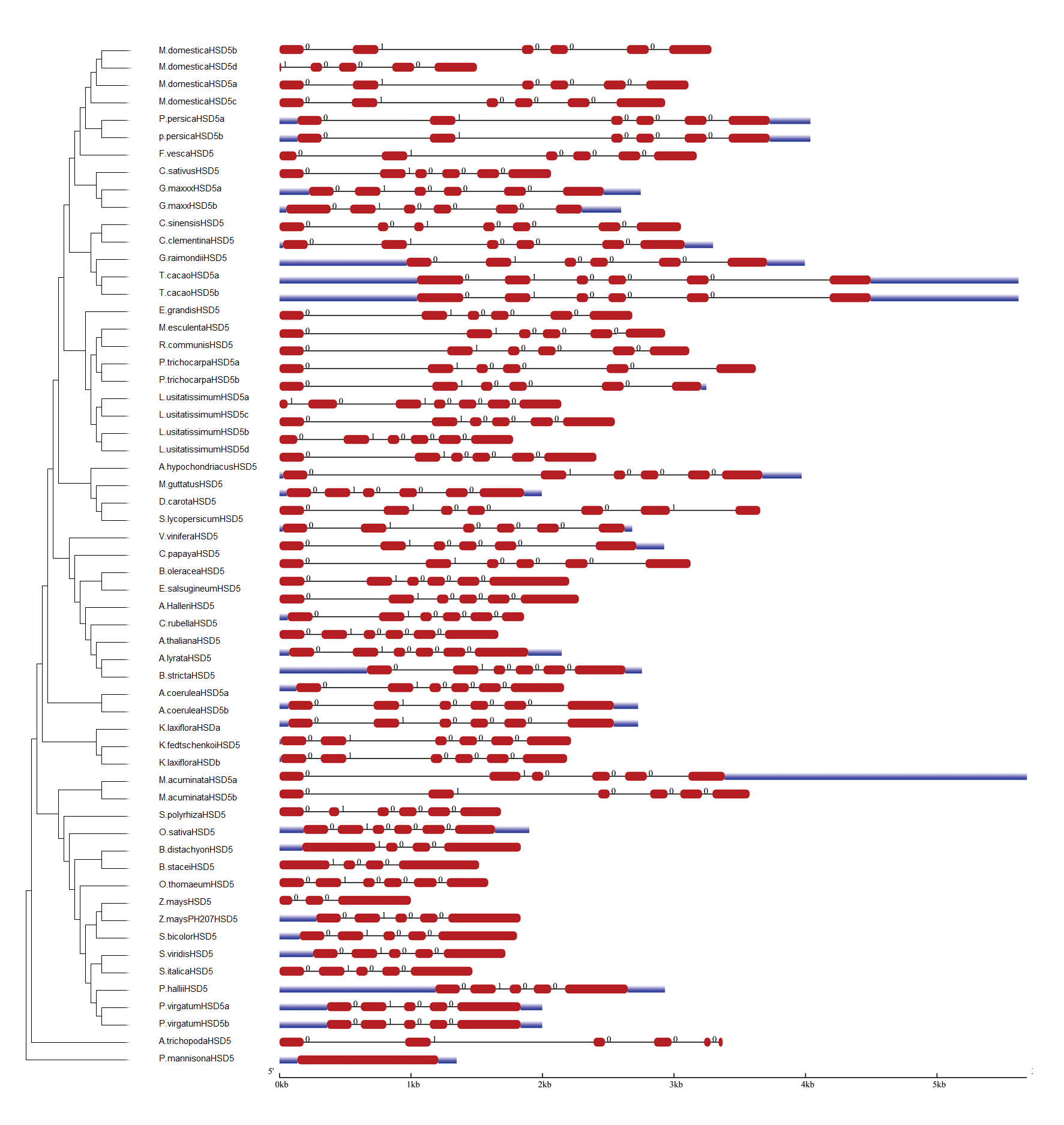


**B**


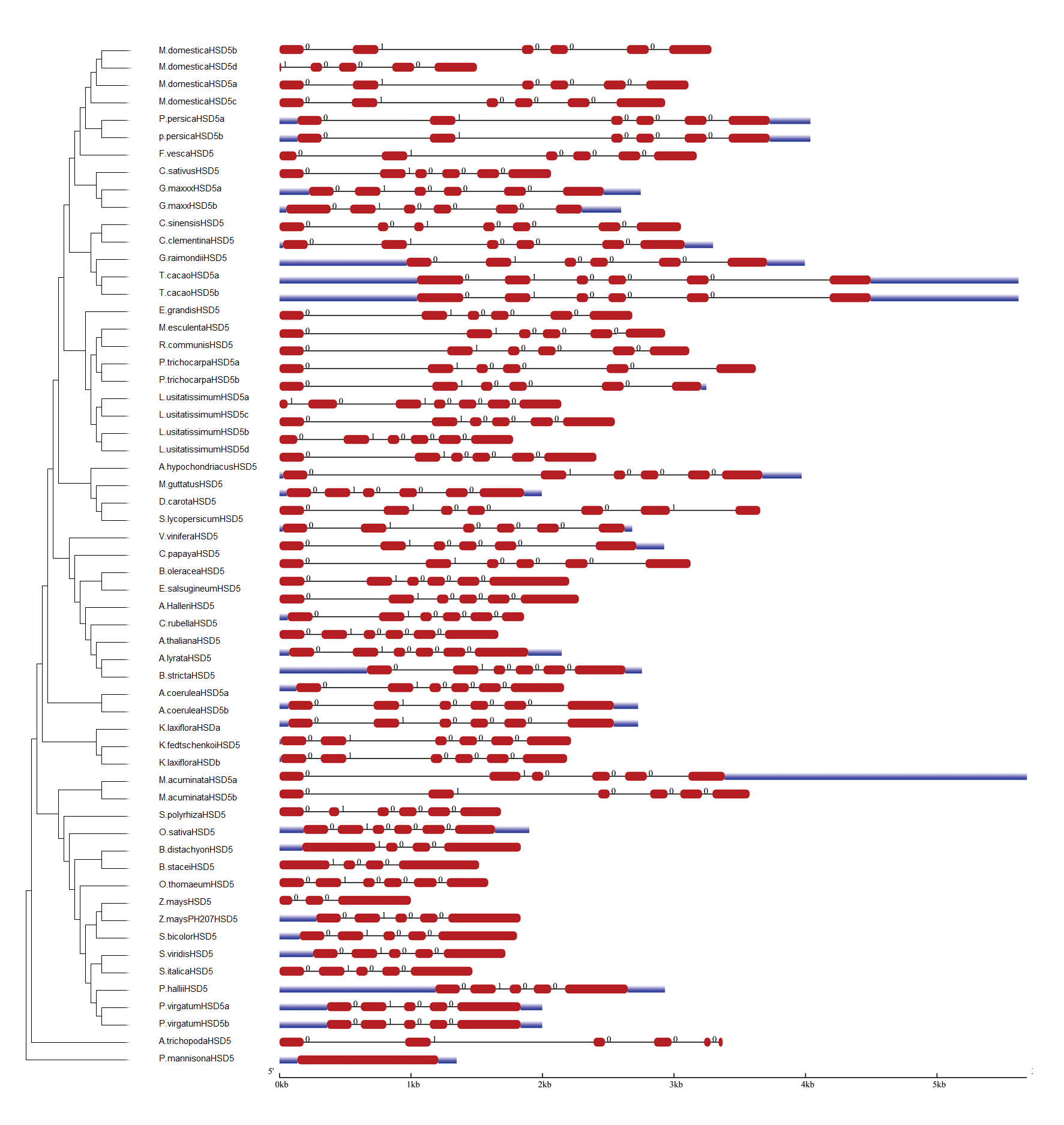


**B**

**Supplementary Figure 2.** Intron phase analysis of HSDs. **(B)** Gene structures of HSD5s. The red blocks represent exon, the black lines represent intron and blue blocks represent untranslated regions. Phases of introns: **0** means introns between two consecutive codons, **1** means intron between first and second nucleotide of a codon and **2** means intron between second and third nucleotide of a codon.


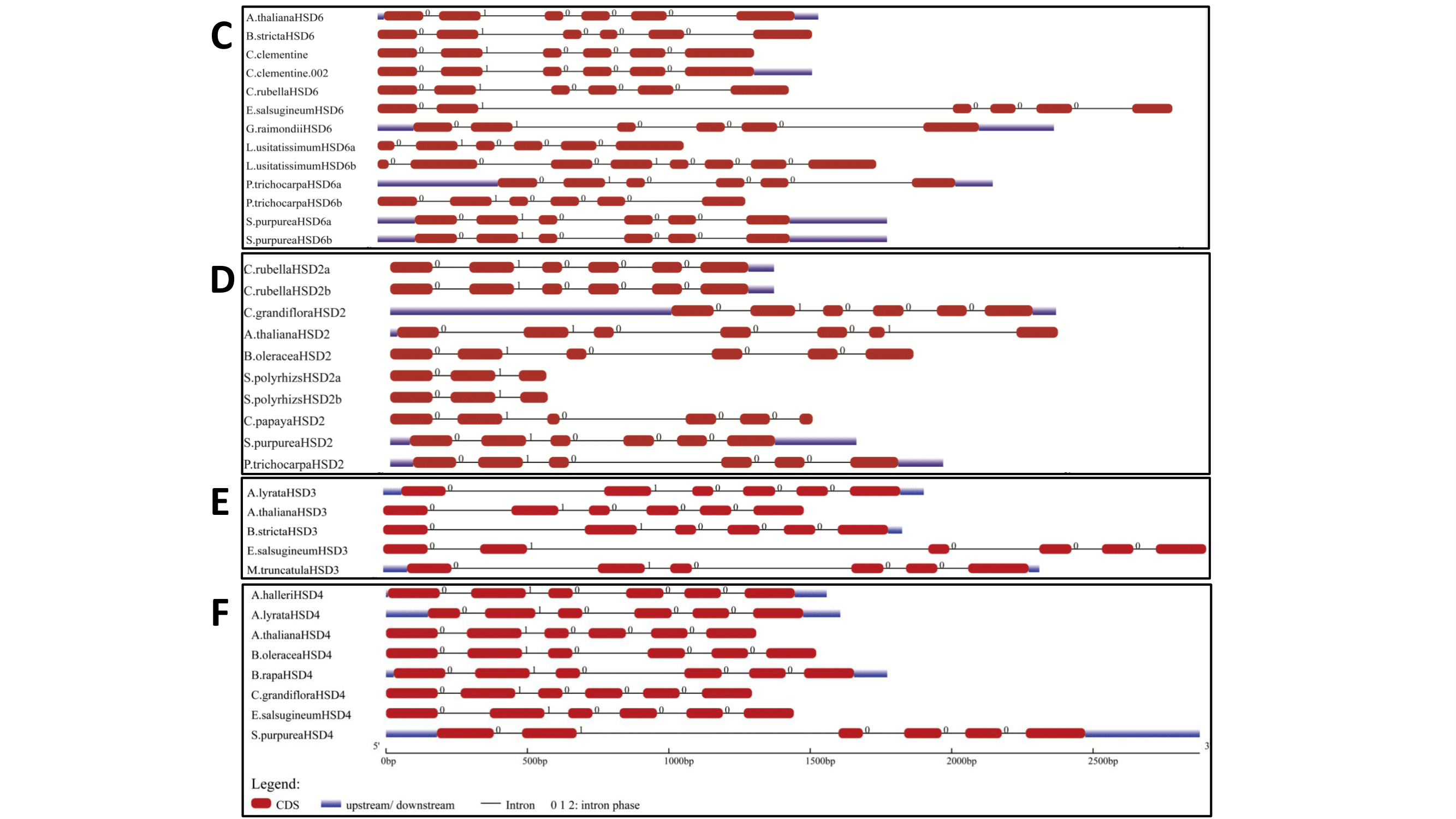
**Supplementary Figure 3.** Intron phase analysis of HSDs. **(C)** Gene structures of HSD6s. **(D)** Gene structures of HSD2s. **(E)** Gene structures of HSD3s. **(F)** Gene structures of HSD4s. The red blocks represent exon, the black lines represent intron and blue blocks represent untranslated regions. Phases of introns: **0** means introns between two consecutive codons, **1** means intron between first and second nucleotide of a codon and **2** means intron between second and third nucleotide of a codon.


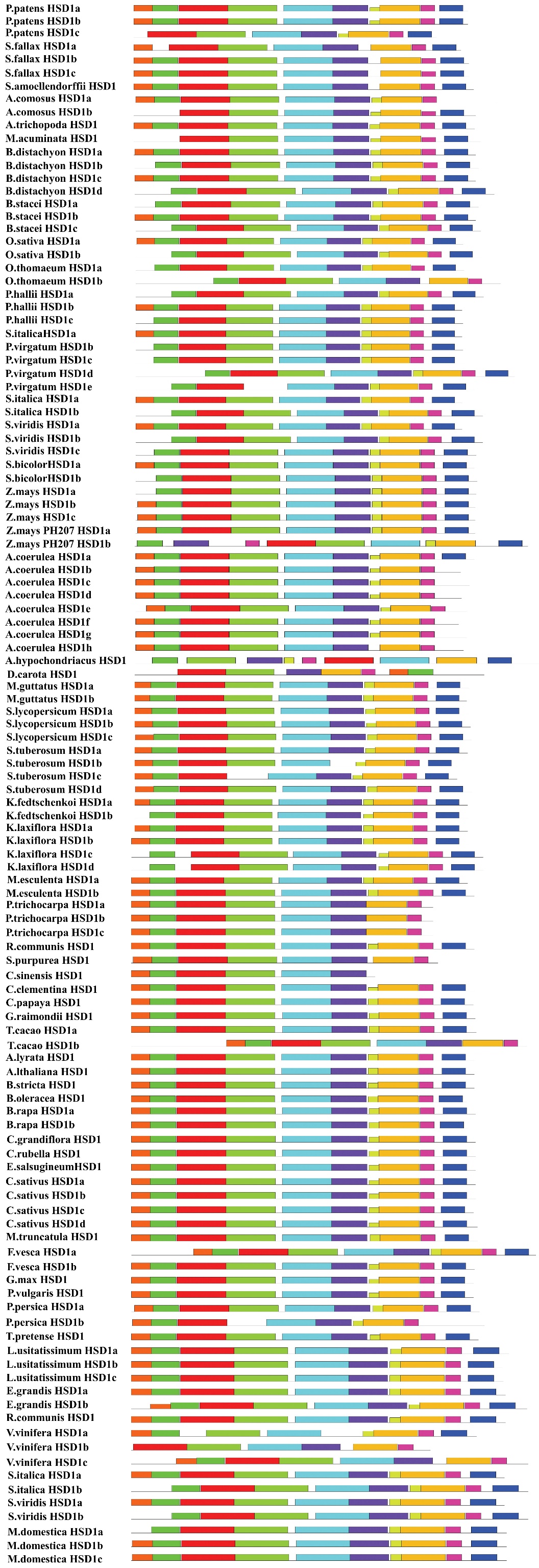

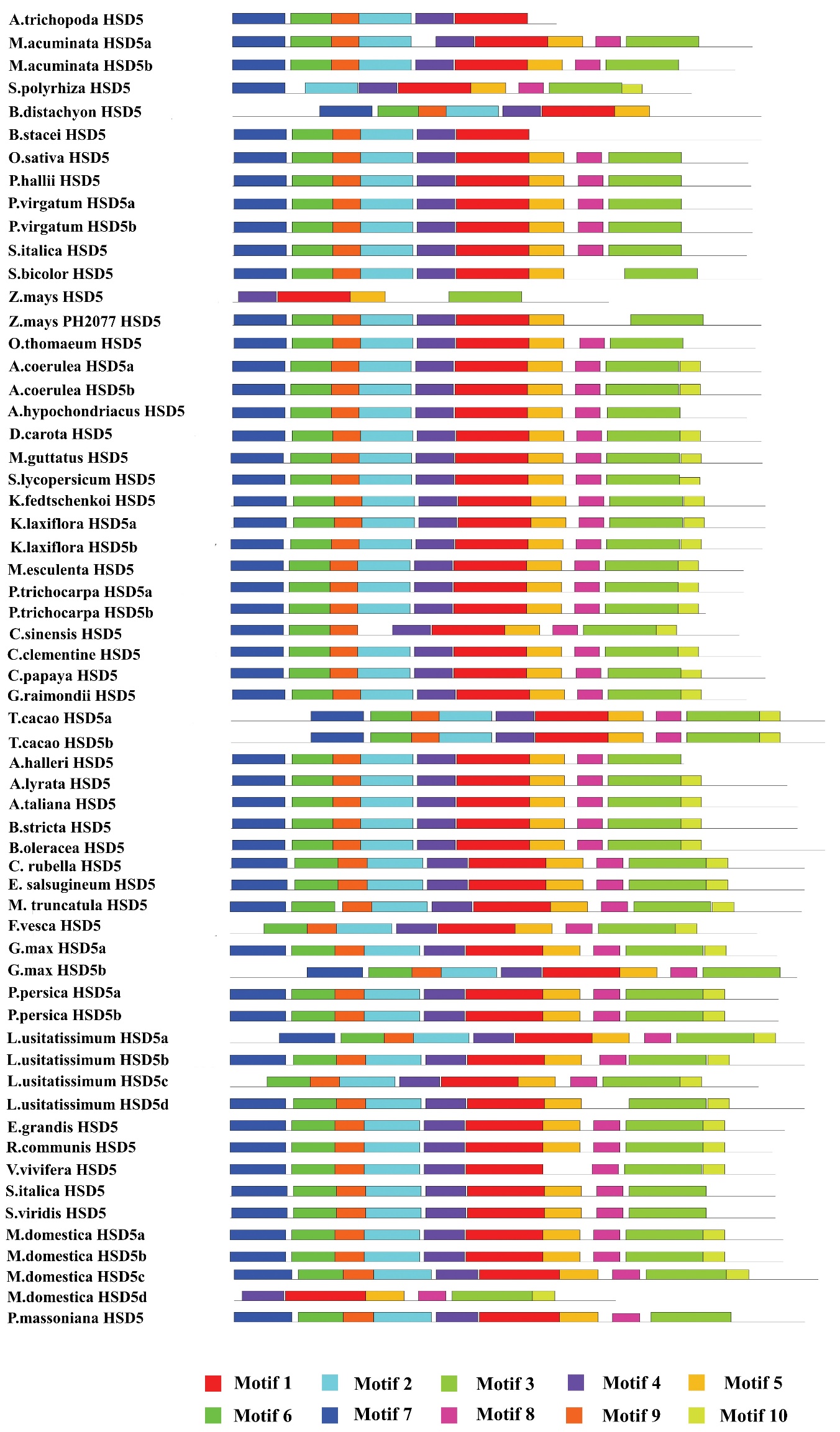

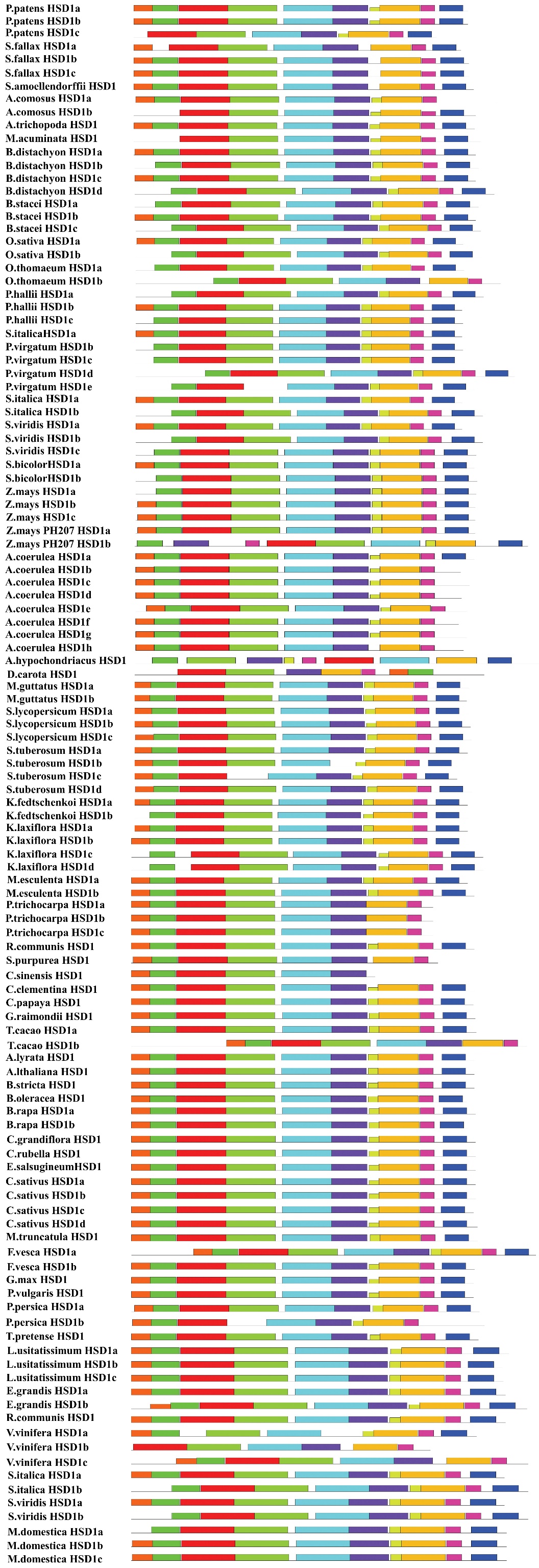

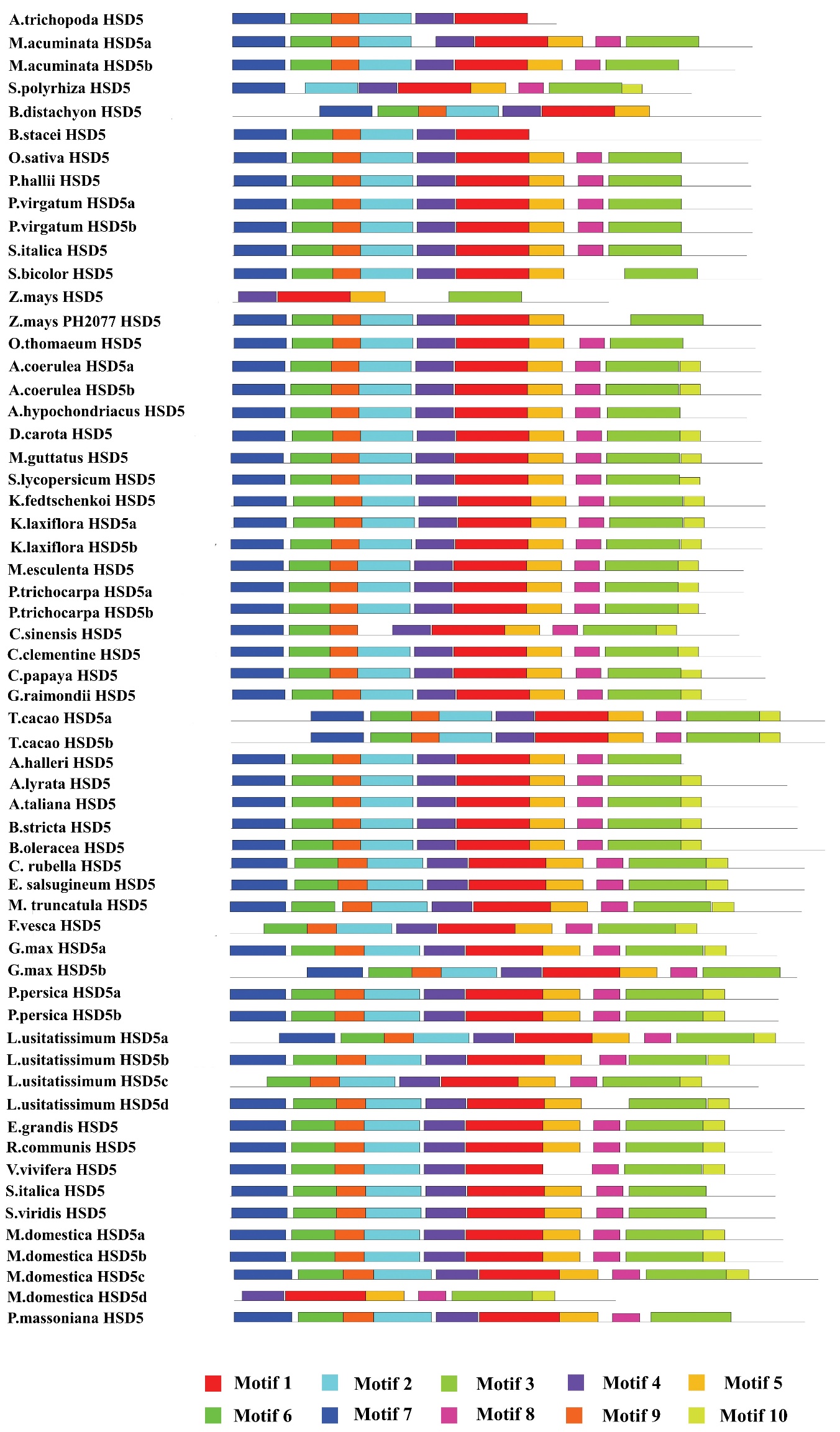


**A**

**B**

**A**

**B**

**Supplementary Figure 2.** Conserved motif analysis. **(A)** Motif patterns of representative sequences of HSD1. **(B)** Motif patterns of representative sequences of HSD5s.


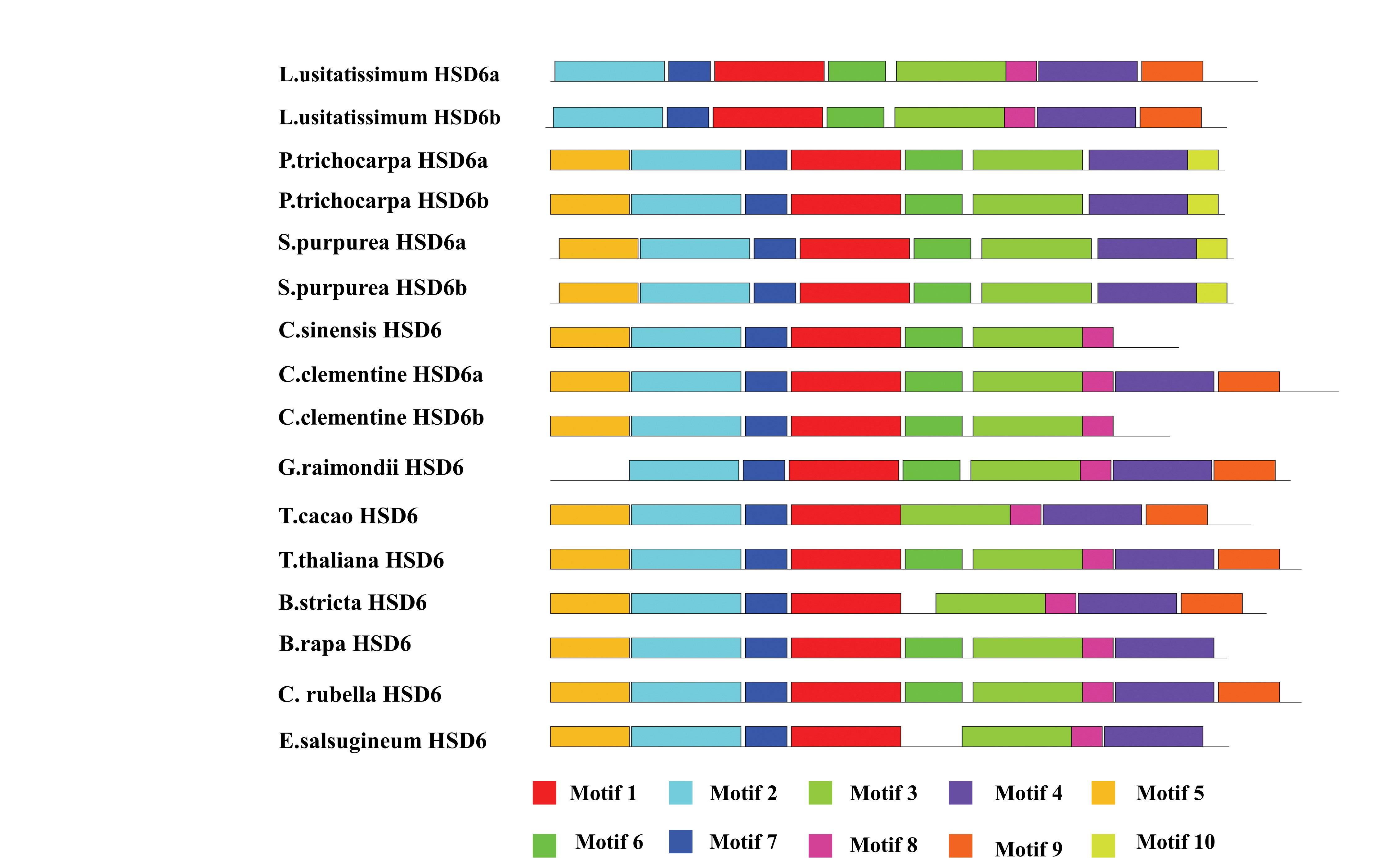

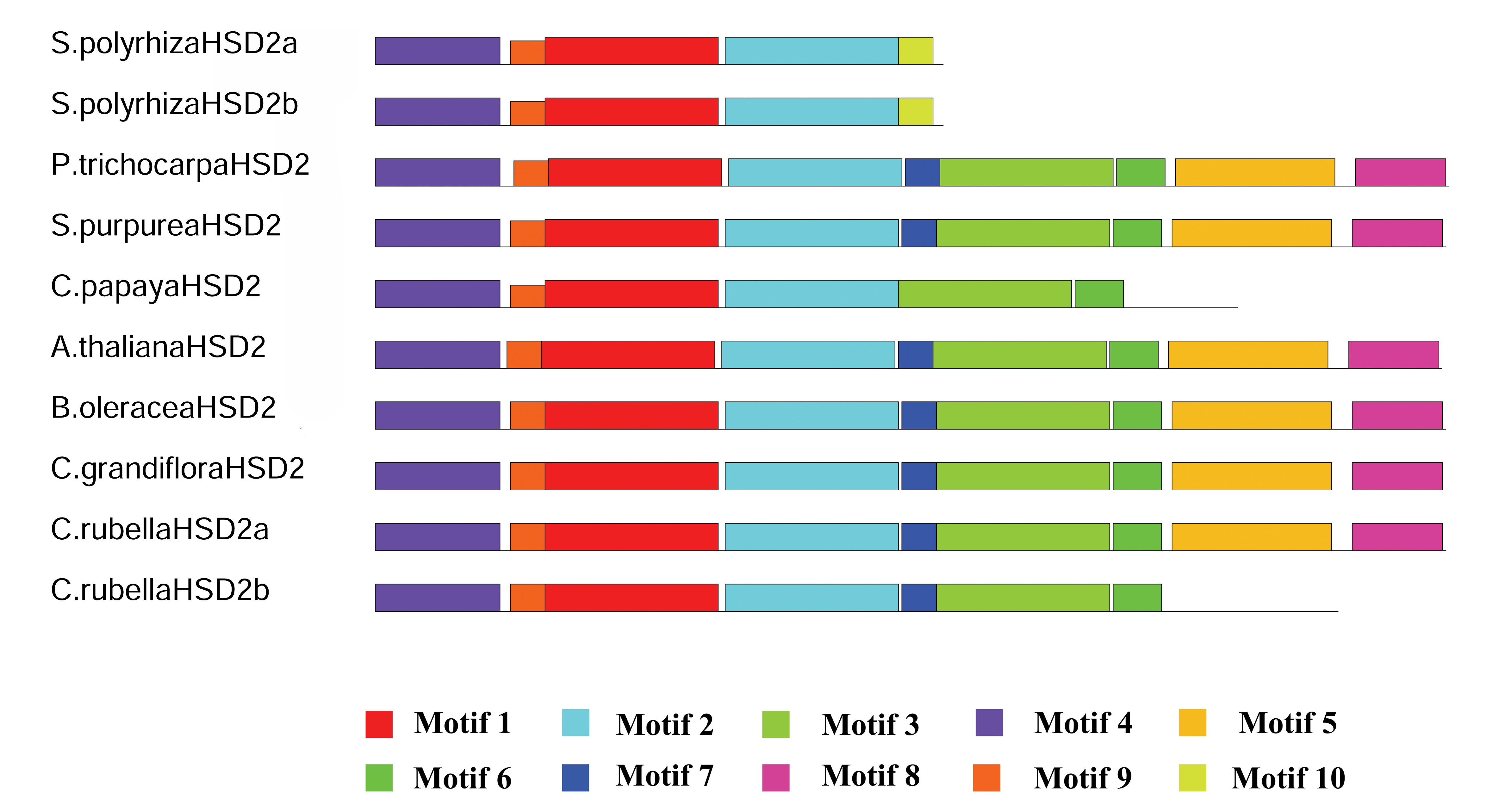

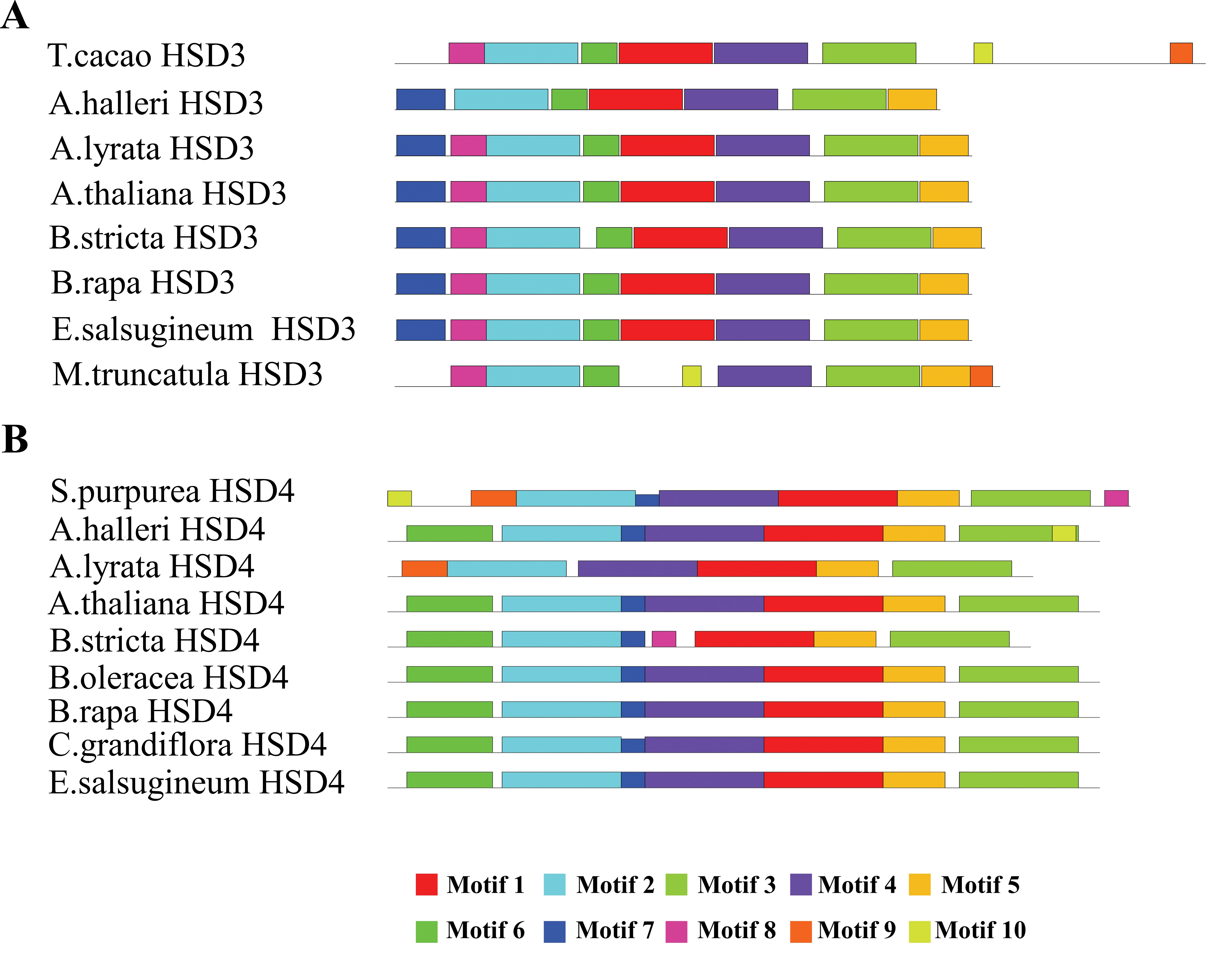

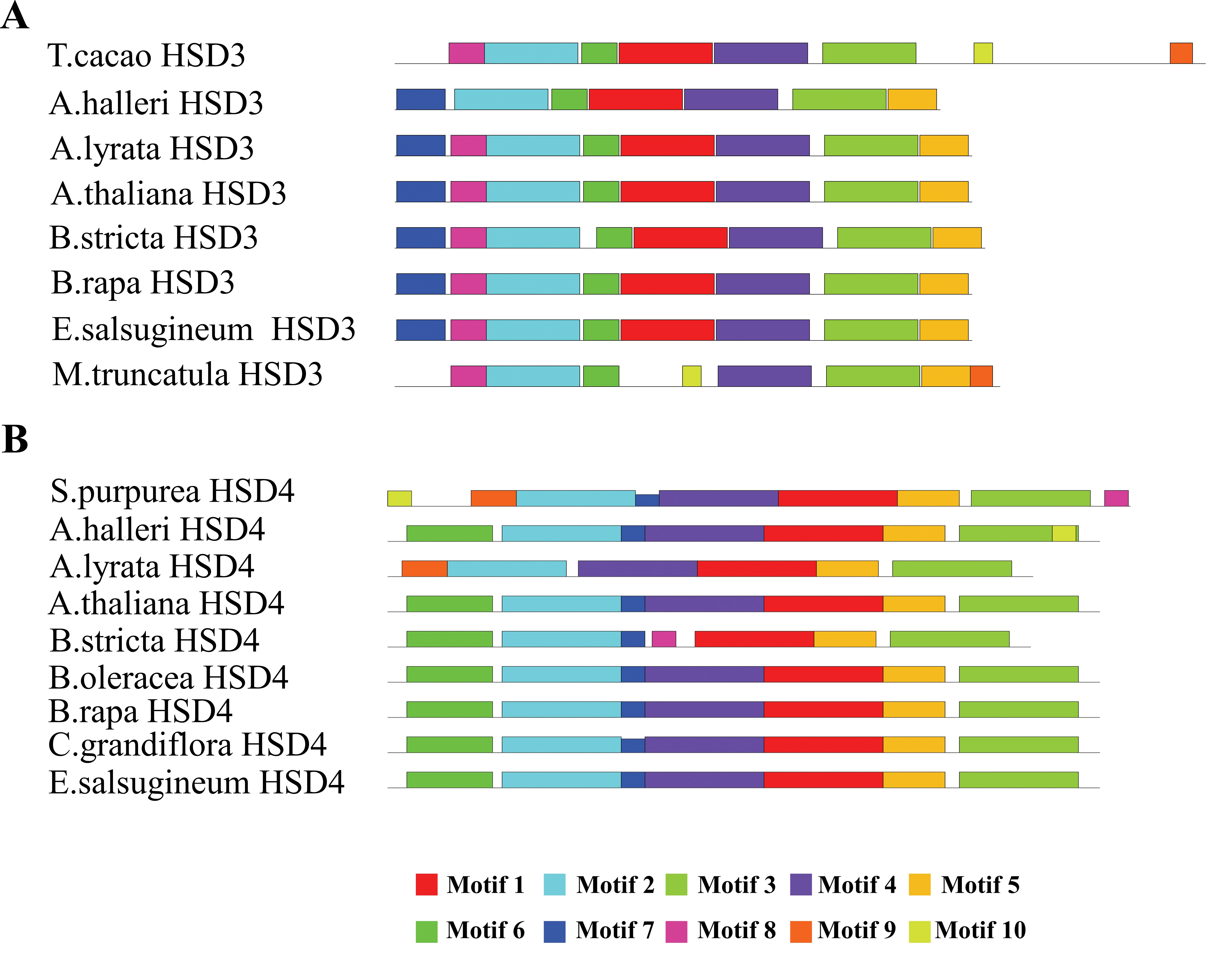


**C**

**D**

**E**

**F**

**Supplementary Figure 2.** Conserved motif analysis. **(C)** Motif patterns of representative sequences of HSD6. **(D)** Motif patterns of representative sequences of HSD2s. **(E)** Motif patterns of representative sequences of HSD3. **(F)** Motif patterns of representative sequences of HSD4. The conserved domains were identified using MEME web server (http://meme-suite .org/tools /meme/). The ten identified motifs were represented in different colors.

## Supplementary Tables

## Supplementary Table 1(A). Identification of HSD1s in plant species

| **Phylum** | **Species** | **Gene Name** | **Gene ID** | **Identification results** |
| --- | --- | --- | --- | --- |
| **Moss** |  |  |  |  |
|  | *Volvex carteri* | V. carteri-HSD-like |  |  |
|  | *Physcomitrella patens* |  |  |  |
|  |  | P. patens HSD1a | Pp3c20_12760V3.3 | Identified |
|  |  | P. patens HSD1b | Pp3c20_12760V3.1 | Identified |
|  |  | P. patens HSD1c | Pp3c20_12760V3.2 | Identified |
|  | *Sphagnum fallax* |  |  |  |
|  |  | S. fallax HSD1a | Sphfalx0023s0058.1 | Identified |
|  |  | S. fallax HSD1b | Sphfalx0075s0092.1 | Identified |
|  |  | S. fallax HSD1c | Sphfalx0075s0092.2 | Identified |
| **Ferns** |  |  |  |  |
|  | *Selaginell amoellendorffii* | S. amoellendorffii HSD1 | 87233 | Identified |
| **Monocots** |  |  |  |  |
|  | *Ananas comosus* | A. comosus HSD1a | Aco016052.1 | Identified |
|  |  | A. comosus HSD1b | Aco003650.1 |  |
|  | *Amborella trichopoda* | A. trichopoda HSD1 | evm_27. model. AmTr_v1.0_scaffold00016.215 | Identified |
|  | *Musa acuminate* | M. acuminata HSD1. | GSMUA_Achr6T12220_001 | Identified |
|  | *Brachypodium distachyon* | B. distachyon HSD1a | Bradi4g07670.3 | Identified |
|  |  | B. distachyon HSD1b | Bradi5g08297.1 | Identified |
|  |  | B. distachyon HSD1c | Bradi4g07670.2 | Identified |
|  |  | B. distachyon HSD1d | Bradi5g08290.1 | Identified |
|  | *Brachypodium stacei* | B. stacei HSD1a | Brast09G060500.1 | Identified |
|  |  | B. stacei HSD1b | Brast10G087200.1 | Identified |
|  |  | B. stacei HSD1c | Brast09G060400.1 |  |
|  | *Oryza sativa* | O. sativa HSD1a | LOC_Os12g27830.1 | Identified |
|  |  | O. sativa HSD1b | LOC_Os04g32070.1 |  |
|  | Oropetium thomaeum | O. thomaeum HSD1a | Oropetium_20150105_0 8783A | Identified |
|  | *Panicum hallii* | P. hallii HSD1a | Pahal.G00868.1 | Identified |
|  |  | P. hallii HSD1b | Pahal.C03801.1 | Identified |
|  | *Panicum virgatum* | P. virgatum HSD1a | Pavir.Ca02179.1 | Identified |
|  |  | P. virgatum HSD1b | Pavir.J20925.1 | Identified |
|  |  | P. virgatum HSD1c | Pavir.Ga02102.1 | Identified |
|  | *Setaria italica* | S. italica HSD1a | Seita.3G316100.1 | Identified |
|  |  | S. italica HSD1b | Seita.7G076000.1 | Identified |
|  | *Setaria viridis* | S. viridis HSD1a | Sevir.3G326600.1 | Identified |
|  |  | S. viridis HSD1b | Sevir.7G082500.1 | Identified |
|  | *Sorghum bicolor* | S. bicolorHSD1 | Sobic.002G393100.1 | Identified |
|  | *Zea mays* | Z. mays HSD1a | GRMZM2G108338_T01 | Identified |
|  |  | Z. mays HSD1b | GRMZM2G006119_T01 | Identified |
|  | *Zea mays PH207* | Z. mays PH207 HSD1 | Zm00008a029486_T01 | Identified |
| **Dicots** |  |  |  |  |
|  | *Aquilegia coerulea* | A. coerulea HSD1a | Aqcoe3G182300.1 | Identified |
|  |  | A. coerulea HSD1b | Aqcoe5G348000.1 | Identified |
|  |  | A. coerulea HSD1c | Aqcoe5G349000.1 | Identified |
|  |  | A. coerulea HSD1d | Aqcoe5G348100.1 | Identified |
|  |  | A. coerulea HSD1e | Aqcoe5G348300.1 | Identified |
|  |  | A. coerulea HSD1f | Aqcoe5G363400.4 | Identified |
|  |  | A. coerulea HSD1g | Aqcoe5G363400.3 | Identified |
|  |  | A. coerulea HSD1h | Aqcoe5G348700.1 | Identified |
|  | *Amaranthus hypochondriacus* | A. hypochondriacus HSD1 | AH017252-RA | Identified |
|  | *Daucus carota* | D. carota HSD1 | DCAR_007506 | Identified |
|  | *Mimulus guttatus* | M. guttatus HSD1 | Migut.I00555.1 | Identified |
|  | *Solanum lycopersicum* | S. lycopersicum HSD1a | Solyc06g072670.2.1 | Identified |
|  |  | S. lycopersicum HSD1b | Solyc03g097440.2.1 | Identified |
|  | *Solanum tuberosum* | S. tuberosum HSD1a | PGSC0003DMT400069254 | Identified |
|  |  | S. tuberosum HSD1b | PGSC0003DMT400092688 | Identified |
|  | *Kalanchoe fedtschenkoi* | K. fedtschenkoi HSD1a | Kaladp0056s0020.1 | Identified |
|  |  | K. fedtschenkoi HSD1b | Kaladp0068s0105.1 | Identified |
|  | *Kalanchoe laxiflora* | K. laxiflora HSD1a | Kalax.0226s0034.1 | Identified |
|  |  | K. laxiflora HSD1b | Kalax.0347s0012.1 | Identified |
|  |  | K. laxiflora HSD1c | Kalax.0054s0092.1 | Identified |
|  |  | K. laxiflora HSD1d | Kalax.0925s0010.1 | Identified |
|  | *Manihot esculenta* | M. esculanta HSD1a | Manes.06G121600.1 | Identified |
|  |  | M. esculanta HSD1b | Manes.06G121900.1 | Identified |
|  | *Populus trichocarpa* | P. trichocarpa HSD1a | Potri.015G100000.1 | Identified |
|  |  | P. trichocarpa HSD1b | Potri.015G099900.1 | Identified |
|  |  | P. trichocarpa HSD1c | Potri.012G101900.1 | Identified |
|  | *Ricinus communis* | R. communis HSD1 | 30147.m014465 | Identified |
|  | *Salix purpurea* | S. purpurea HSD1 | apurV1A.0306s0330.1 | Identified |
|  | *Citrus sinensis* | C. sinensis HSD1 | >orange1.1g044010m | Identified |
|  | *Citrus clementina* | C. clementina HSD1 | Ciclev10031969m | Identified |
|  | *Carica papaya* | C. papaya HSD1 | evm. model. supercontig_4.164 | Identified |
|  | *Gossypium raimondii* | G. raimondii HSD1 | Gorai.003G118700.1 | Identified |
|  | *Theobroma cacao* | T. cacao HSD1a | Thecc1EG014683t1 | Identified |
|  |  | T. cacao HSD1b | Thecc1EG014684t1 |  |
|  | *Arabidopsis lyrata*  *Arabidopsis thaliana* | A. lyrata HSD1  A. thaliana HSD1 | AL8G24860.t1  At5g50600 | Identified |
|  | *Boechera stricta* | B. stricta HSD1 | Bostr.15774s0209.1 | Identified |
|  | *Brassica oleracea* | B. oleracea HSD1 | Bol015090 | Identified |
|  | *Brassica rapa* | B. rapa HSD1a | Brara.B01581.1 | Identified |
|  |  | B. rapa HSD1b | Brara.C02545.1 | Identified |
|  | *Capsella grandiflora* | C. grandiflora HSD1 | Cagra.0117s0011.1 | Identified |
|  | *Capsella rubella* | C. rubella HSD1 | Carubv10028347m | Identified |
|  | *Eutrema salsugineum* | E. salsugineum HSD1 | Thhalv10015475m | Identified |
|  | *Cucumis sativus* | C. sativus HSD1a | Cucsa.134850.1 | Identified |
|  |  | C. sativus HSD1b | Cucsa.134860.1 | Identified |
|  |  | C. sativus HSD1c | Cucsa.134840.1 | Identified |
|  | *Medicago truncatula* | M. truncatula HSD1 | Medtr8g096620.1 | Identified |
|  | *Fragaria vesca* | F. vesca HSD1a | mrna06286.1-v1.0-hybrid | Identified |
|  |  | F. vesca HSD1b | mrna06490.1-v1.0-hybrid | Identified |
|  | *Glycine max* | G. max HSD1 | Glyma.08G010800.2 | Identified |
|  | *Phaseolus vulgaris* | P. vulgaris HSD1 | Phvul.002G285000.1 | Identified |
|  | *Prunus persica* | P. persica HSD1a | Prupe.5G148100.1 | Identified |
|  |  | P. persica HSD1b | Prupe.5G147800.1 | Identified |
|  | *Trifolium pratense* | T. pretense HSD1 | Tp57577_TGAC_v2_mRNA13168 | Identified |
|  | *Linum usitatissimum* | L. usitatissimum HSD1a | Lus10032556 | Identified |
|  |  | L. usitatissimum HSD1b | Lus10043187 | Identified |
|  |  | L. usitatissimum HSD1c | Lus10031448 | Identified |
|  | *Eucalyptus grandis* | E. grandis HSD1 | Eucgr.B00089.1 | Identified |
|  | *Vitis vinifera* | V. vinifera HSD1a | GSVIVT01008578001 | Identified |
|  |  | V. vinifera HSD1b | GSVIVT01000914001 | Identified |
|  |  | V. vinifera HSD1c | GSVIVT01000915001 | Identified |
|  | *Malus domestica* | M. domestica HSD1a | MDP0000133528 | Identified |
|  |  | M. domestica HSD1b | MDP0000133527 | Identified |
|  |  | M. domestica HSD1c | MDP0000906098 | Identified |
| **Mammalian** |  |  |  |  |
|  | *Homo sapiens* | H. sapiens HSD1 | Protein ID KAI2521299.1 | Identified |
|  | *Mus musculus* | Mus musculus HSD1 | Sequence ID-NP_001038216.1 | Identified |

**Supplementary Table 1(B).** Identification of HSD5s in plant species

| **Phylum** | **Species** | **Gene Name** | **Gene ID** | **Identification results** |
| --- | --- | --- | --- | --- |
| **Gymnosperms** | Pinus massoniana | P. massoniana HSD5 | [KT731102](https://www.ncbi.nlm.nih.gov/nuccore/1001229721) | identified |
| **Monocots** |  |  |  |  |
|  | Ananas comosus | A. comosus HSD5 | Aco004791.1 | Lack of start codon |
|  | *Amborella trichopoda* | A. trichopoda HSD5 | evm_27. model. AmTr_v1.0_scaffold00099.13 | Identified |
|  | *Musa acuminata* | M. acuminata HSD5a | GSMUA_AchrUn_randomT21270_001 | Identified |
|  |  | M. acuminata HSD5b | SMUA_Achr7T02980_001 | Identified |
|  | *Spirodela polyrhiza* | S. polyrhiza HSD5 | Spipo4G0078100 | Identified |
|  | *Zostera marina* | Z. marina ABI3 | Zosma63g00240.1 | Identified |
|  | *Brachypodium distachyon* | B. distachyon HSD5 | Bradi1g02107.3 | Identified |
|  | *Brachypodium stacei* | B. stacei HSD5 | Brast02G378800.1 | Identified |
|  | *Oryza sativa* | O. sativa HSD5 | LOC_Os03g62590.1 | Identified |
|  | *Panicum hallii* | P. hallii HSD5 | Pahal.I01168.1 | Identified |
|  | *Panicum virgatum* | P. virgatum HSD5a | Pavir.Ia00021.1 | Identified |
|  |  | P. virgatum HSD5b | Pavir.Ia00021.2 | Identified |
|  | *Setaria italic* | S. italica HSD5 | Seita.9G017000.1 | Identified |
|  | *Setaria viridis* | S. viridis HSD5 | Sevir.9G016700.1 | Identified |
|  | *Sorghum bicolor* | S. bicolor HSD5 | Sobic.001G017500.1 | Identified |
|  | *Zea mays* | Z. mays HSD5 | AC217358.3_FGT007 | Identified |
|  | *Zea mays PH207* | Z. mays PH207 HSD5 | Zm00008a005765_T01 | Identified |
|  | *Oropetium thomaeum* | O. thomaeum HSD5 | Oropetium_20150105_25859A | Identified |
| **Dicots** |  |  |  |  |
|  | *Aquilegia coerulea* | A. coerulea HSD5a | Aqcoe6G008700.1 | Identified |
|  | *Aquilegia coerulea* | A. coerulea HSD5b | Aqcoe6G008700.2 | Identified |
|  | *Amaranthus hypochondriacus* | A. hypochondriacus HSD5 | AH008604-RA | Identified |
|  | *Daucus carota* | D. carota HSD5 | DCAR_003515 | Identified |
|  | *Solanum lycopersicum* | S. lycopersicum HSD5 | Solyc06g064650.2.1 | Identified |
|  | *Kalanchoe fedtschenkoi* | K. fedtschenkoi HSD5 | Kaladp0057s0049.1 | Identified |
|  | *Kalanchoe laxiflora* | K. laxiflora HSD5a | Kalax.0535s0008.1 | Identified |
|  |  | K. laxiflora HSD5b | Kalax.0127s0013.1 | Identified |
|  | *Manihot esculenta* | M. esculenta HSD5 | Manes.18G119600.1 | Identified |
|  | *Populus trichocarpa* | P. trichocarpa HSD5a | Potri.019G073200.1 | Identified |
|  | *Populus trichocarpa* | P. trichocarpa HSD5b | Potri.013G100200.1 | Identified |
|  | *Salix purpurea* | S. purpurea ABI3 | SapurV1A.0937s0080.1 | Identified |
|  | *Citrus sinensis* | C. sinensis HSD5 | orange1.1g039397m | Identified |
|  | *Citrus clementina* | C. clementina HSD5 | Ciclev10020750m | Identified |
|  | *Carica papaya* | C. papaya HSD5 | evm. model. supercontig_21.62 | Identified |
|  | *Gossypium raimondii* | G. raimondii HSD5 | Gorai.009G249100.1 | Identified |
|  | *Theobroma cacao* | T. cacao HSD5a | Thecc1EG036223t2 | Identified |
|  | *Theobroma cacao* | T. cacao HSD5b | Thecc1EG036223t1 | Identified |
|  | *Arabidopsis halleri* | A. Halleri HSD5 | Araha.10723s0003.1 | Lack of stop codon |
|  | *Arabidopsis lyrata* | A. lyrata HSD5 | AL6G43740.t1 | Identified |
|  | *Arabidopsis thaliana* | A. thaliana HSD5 | AT4G10020.1 | Identified |
|  | *Boechera stricta* | B. stricta HSD5 | Bostr.25463s0321.1 | Identified |
|  | *Brassica oleracea* | B. oleracea HSD5 | Bol025657 | Identified |
|  | *Brassica rapa FPSC* | B. rapa HSD5 | Brara.C02625.1 | Identified lack domain |
|  | *Capsella grandiflora* |  | Cagra.7220s0001.1 | Identified but Lack ATG |
|  | *Capsella rubella* | C. rubella HSD5 | Carubv10003016m | Identified |
|  | *Eutrema salsugineum* | E. salsugineum HSD5 | Thhalv10029315m | Identified |
|  | *Cucumis sativus* | C. sativus HSD5 | Cucsa.236970.1 | Identified |
|  | *Medicago truncatula* |  | Medtr5g008850.1 | Identified but lack domain |
|  | *Fragaria vesca* | F. vesca HSD5 | mrna27063.1-v1.0-hybrid | Identified |
|  | *Glycine max* | G. max HSD5a | Glyma.01G227900.1 | Identified |
|  |  | G. max HSD5b | Glyma.11G015100.1 | Identified |
|  | *Phaseolus vulgaris* | P. vulgaris HSD5 | Phvul.008G122100 | Identified |
|  | *Prunus persica* | P. persica HSD5a | Prupe.1G546200.1 | Identified |
|  |  | P. persica HSD5b | Prupe.1G546200.2 | Identified |
|  | *Trifolium pratense* | T. pratense HSD5 | Tp57577_TGAC_v2_mRNA39243 | Identified but lack domain |
|  | *Linum usitatissimum* | L. usitatissimum HSD5a | Lus10041045 | Identified |
|  | *Linum usitatissimum* | L. usitatissimum HSD5b | Lus10009467 | Identified |
|  | *Linum usitatissimum* | L. usitatissimum HSD5c | Lus10006178 | Identified |
|  | *Linum usitatissimum* | L. usitatissimum HSD5d | Lus10001280 | Identified |
|  | *Eucalyptus grandis* | E. grandis HSD5 | Eucgr.F02966.1 | Identified |
|  | *Ricinus communis* | R. communis HSD5 | 29938.m000612 | Identified |
|  | *Vitis vinifera* | V. vinifera HSD5 | GSVIVT01036430001 | Identified |
|  | *Setaria italica* | S. italica HSD5 | Seita.9G017000.1 | Identified |
|  | *Setaria viridis* | S. viridis HSD5 | Sevir.9G016700.1 | Identified |
|  | *Malus domestica* | M. domestica HSD5a | MDP0000315759 | Identified |
|  |  | M. domestica HSD5b | MDP0000204529 | Identified |
|  |  | M. domestica HSD5c | MDP0000265006 | Identified |
|  |  | M. domestica HSD5d | MDP0000124544 | Identified |

**Supplementary Table 1(C).** Identification of HSD6s in plant species

| **Phylum** | **Species** | **Gene Name** | **Gene ID** | **Identification results** |
| --- | --- | --- | --- | --- |
| **Dicots** |  |  |  |  |
|  | *Aquilegia coerulea* |  | Aqcoe3G182300.1 | Identified as 3rd |
|  | *Amaranthus hypochondriacus* |  | AHYPO_004692-RA | Identified as 3rd |
|  | *Daucus carota* |  | DCAR_007506 | Identified as 3rd |
|  | *Mimulus guttatus* |  | Migut.I00556.1 | Identified as 3rd |
|  | *Solanum lycopersicum* |  | Solyc07g052270.2.1 | Identified as 3rd |
|  | *Solanum tuberosum* |  | PGSC0003DMT400074514 | Identified as 3rd |
|  | *Kalanchoe fedtschenkoi* |  | Kaladp0056s0020.1 | Identified as 3rd |
|  | Kalanchoe laxiflora |  | Kalax.0226s0034.1 | Identified as 3rd |
|  | Eucalyptus grandis |  | Eucgr.K02704.1 | Identified as 3rd |
|  | *Manihot esculenta* |  | Manes.06G121900.1 | Identified as 3rd |
|  | Vitis vinifera |  | GSVIVT01000914001 | Identified as 3rd |
|  | *Populus trichocarpa* | P. trichocarpa HSD6a | Potri.015G099900.1 | Identified |
|  |  | P. trichocarpa HSD6b | Potri.012G101900.1 | Identified |
|  | *Salix purpurea* | S. purpurea HSD6ba | SapurV1A.0168s0530.1 | Identified |
|  | *Salix purpurea* | S. purpurea HSD6b | SapurV1A.0168s0530.1 | Identified |
|  | *Citrus clementine* | C. clementine HSD6a | Ciclev10031927m | identified |
|  | *Citrus clementine* | C. clementine HSD6b | Ciclev10032335m | identified |
|  | *Citrus sinensis* | C. sinensis HSD6 | orange1.1g042560m (primary) | Lack of stop codon |
|  | *Carica papaya* |  | evm. model. supercontig_4.164 | Identified as 3rd |
|  | *Gossypium raimondii* | G. raimondii HSD6 | Gorai.001G056800.1 | identified |
|  | *Arabidopsis lyrata* |  | AL8G24860.t1 | Identified as 3rd |
|  | *Arabidopsis thaliana* | A. thaliana HSD6 | At5g50770 | Identified |
|  | *Boechera stricta* | B. stricta HSD6 | Bostr.15774s0215.1 | Identified |
|  | *Brassica oleracea* |  | Bol015090 | Identified as 3rd |
|  | *Brassica rapa* |  | Brara.B01579.1 | identified |
|  | *Theobroma cacao* | T. cacao HSD6 | Thecc1EG014686t1 | identified |
|  | *Capsella grandiflora* |  | *Cagra.0117s0011.1* | *Identified as 3rd* |
|  | *Capsella rubella* | C. rubella HSD6 | Carubv10028440m | Identified |
|  | *Eutrema salsugineum* | E. salsugineum HSD6 | Thhalv10015936m | Identified |
|  | *Cucumis sativus* |  | Cucsa.134860.1 | Identified as 3rd |
|  | *Medicago truncatula* |  | Medtr8g096620.1 | Identified as 3rd |
|  | *Fragaria vesca* |  | mrna06490.1-v1.0-hybrid | Identified as 3rd |
|  | *Glycine max* |  | Glyma.08G010800.2 | Identified as 3rd |
|  | *Phaseolus vulgaris* |  | Phvul.002G285000.1 | Identified as 3rd |
|  | *Prunus persica* |  | Prupe.5G148100.1 | Identified as 3rd |
|  | *Trifolium pretense* |  | Tp57577_TGAC_v2_mRNA13168 | Identified as 3^rd^ |
|  | *Linum usitatissimum* | L. usitatissimum HSD6a | Lus10022441 | Identified |
|  | *Linum usitatissimum* | L. usitatissimum HSD6b | Lus10016748 | Identified |
|  | *Eucalyptus grandis* |  | Eucgr.K02704.1 | Identified as 3rd |
|  | *Ricinus communis* |  | 30147.m014465 | Identified as 3rd |
|  | *Vitis vinifera* |  | GSVIVT01000914001 | Identified as 3rd |
|  | *Setaria italic* |  | Seita.3G316100.1 | Identified as 3rd |
|  | *Setaria viridis* |  | Sevir.3G326600.1 | Identified as 3rd |
|  | *Malus domestica* |  | MDP0000906098 | Identified as 3rd |

**Supplementary Table 1 (D).** Identification of HSD2s in plant species

| **Phylum** | **Species** | **Gene Name** | **Gene ID** | **Identification results** |
| --- | --- | --- | --- | --- |
| **Monocots** |  |  |  |  |
|  | Spirodela polyrhiza | S. polyrhiza HSD2a | Spipo7G0048100 |  |
|  | Spirodela polyrhiza | S. polyrhiza HSD2b | Spipo10G0040700 |  |
|  | *Brachypodium distachyon* |  | Bradi4g07670.3 | Identified as 4^th^ |
|  | *Brachypodium stacei* |  | Brast10G087200.1 | Identified as 4^th^ |
|  | *Panicum virgatum* |  | Pavir.Ca02179.1 | Identified as 5t^h^ |
|  | *Setaria italic* |  | Seita.3G316100.1 | Identified as 5th |
|  | *Zea mays* |  | GRMZM2G006119_T01 | Identified as 5^th^ |
|  | *Zea mays PH207* |  | Zm00008a029486_T01 | Identified as 5^th^ |
| **Dicots** |  |  |  |  |
|  | *Aquilegia coerulea* |  | Aqcoe5G348300.1 | Identified as 4^th^ |
|  | *Solanum lycopersicum* |  | Solyc03g097440.2.1 | Identified as 5^th^ |
|  | *Solanum tuberosum* |  | PGSC0003DMT400069254 | Identified as 4^th^ |
|  | Brassica oleracea | B. oleracea HSD2 | Bol037225 | Identified |
|  | Salix purpurea | S. purpurea HSD2 | SapurV1A.1018s0020.1 | Identified |
|  | Populus trichocarpa | P. trichocarpa HSD2 | Potri.012G102000.1 | Identified |
|  | *Carica papaya* | C. papaya HSD2 | evm. model. supercontig_4.163 | Identified |
|  | *Arabidopsis thaliana* | A. thaliana HSD2 | At3g47350 | Identified |
|  | Capsella grandiflora | C. grandiflora HSD2 | Cagra.4069s0001.1 | Identified |
|  | *Capsella rubella* | C. rubella HSD2a | Carubv10017691m | Identified |
|  | *Capsella rubella* | C. rubella HSD2b | Carubv10017803m | Identified |
|  | *Trifolium pretense* |  | Tp57577_TGAC_v2_mRNA13168 | Identified as 4^th^ |
|  | *Eucalyptus grandis* |  | Eucgr.B00089.1 | Identified as 4^th^ |
|  | *Vitis vinifera* |  | GSVIVT01000915001 | Identified as 4^th^ |

**Supplementary Table 1(E).** Identification of HSD3s in plant species

| **Phylum** | **Species** | **Gene Name** | **Gene ID** | **Identification results** |
| --- | --- | --- | --- | --- |
| **Dicots** |  |  |  |  |
|  | *Daucus carota* |  | DCAR_007506 | Identified at 4th |
|  | *Mimulus guttatus* |  |  | Identified at 5th |
|  | *Citrus sinensis* |  |  | Identified at 3rd |
|  | *Theobroma cacao* | T. cacao HSD3 | Thecc1EG014685t1 (primary) | Identified |
|  | *Arabidopsis halleri* | A. halleri HSD3 | Araha.40071s0002.1 (primary) | Identified |
|  | *Arabidopsis lyrata* | A. lyrata HSD3 | AL5G26530.t1 (primary) | Identified |
|  | *Arabidopsis thaliana* | A. thaliana HSD3 | AT3G47360.1 (primary) | Identified |
|  | *Boechera stricta* | B. stricta HSD3 | Bostr.18473s0233.1 (primary) | Identified |
|  | *Brassica rapa FPSC* | B. rapa FPSC HSD3 | Brara.A02442.1 (primary) | Deleted due to lack of stop codon |
|  | *Eutrema salsugineum* | E. salsugineum HSD3 | Thhalv10011076m (primary) | Identified |
|  | Medicago truncatula | M. truncatula HSD3 | Medtr8g096580.1 | Identified |
|  | *Cucumis sativus* |  |  | Identified at 4th |

**Supplementary Table 1 (F).** Identification of HSD4s in plant species

| **Phylum** | **Species** | **Gene Name** | **Gene ID** | **Identification results** |
| --- | --- | --- | --- | --- |
| **Dicots** |  |  |  |  |
|  | *Aquilegia coerulea* |  | Aqcoe5G348300.1 | Identified as 4^th^ |
|  | *Solanum tuberosum* |  | PGSC0003DMT400074514 | Identified as 5^th^ |
|  | Citrus clementine |  | Ciclev10031969m | Identified as 4^th^ |
|  | Citrus sinensis |  | orange1.1g042560m | Identified as 4^th^ |
|  | Theobroma cacao |  | Thecc1EG014685t1 | Identified as 4^th^ |
|  | Gossypium raimondii |  | Gorai.003G118700.1 | Identified as 4^th^ |
|  | Brassica oleracea | B. oleracea HSD4 | Bol044967 | Identified |
|  | Brassica stricta | B. stricta HSD4 | Bostr.15774s0208.1 | Identified |
|  | Eutrema salsugineum | E. salsugineum HSD4 | Thhalv10014225m | Identified |
|  | Salix purpurea | S. purpurea HSD4 | SapurV1A.0018s0450.1 | Identified |
|  | Brassica Rapa | B. rapa HSD4 | Brara.J00698.1 | Identified |
|  | *Arabidopsis halleri* | A. halleri HSD4 | Araha.4353s0015.1 | Identified |
|  | Arabidopsis lyrata | A. lyrata HSD4 | AL8G24850.t1 | identified |
|  | *Arabidopsis thaliana* | A. thaliana HSD4 | At5g50590 | Identified |
|  | Capsella grandiflora | C. grandiflora HSD4 | Cagra.0117s0010.1 | Identified |
|  | Prunus persica |  | Prupe.5G148000.1 | Identified as 4^th^ |

**Supplementary Table 2.** Potential cis-elements of *Arabidopsis thaliana* HSDs genes predicted by PlantCARE

| **Cis-element** | **Description** | **AtHSD5** | | **AtHSD1** | **AtHSD4** | **AtHSD6** | **AtHSD3** | **AtHSD4a** | **AtHSD4b** | **AtHSD2** |
| --- | --- | --- | --- | --- | --- | --- | --- | --- | --- | --- |
| 5UTR Py-rich stretch | cis-acting element conferring high transcription levels | | 0 | 0 | 0 | 0 | 0 | 2 | 2 | 0 |
| AAGAA-motif | NA | | 0 | 2 | 2 | 0 | 0 | 0 | 0 | 0 |
| 3-AF1 binding site | light responsive element | | 1 | 0 | 0 | 0 | 0 | 0 | 0 | 0 |
| A-box | cis-acting regulatory element | | 0 | 0 | 0 | 0 | 0 | 0 | 0 | 1 |
| AAGAA-motif | NA | | 0 | 0 | 0 | 1 | 0 | 0 | 0 | 1 |
| ABRE | cis-acting element involved in the abscisic acid responsiveness | | 6 | 1 | 1 | 2 | 0 | 0 | 0 | 0 |
| ACE | cis-acting element involved in light responsiveness | | 1 | 1 | 1 | 0 | 0 | 0 | 0 | 1 |
| AE-box | part of a module for light response | | 1 | 0 | 0 | 1 | 2 | 0 | 0 | 2 |
| ARE | cis-acting regulatory element essential for the anaerobic induction | | 2 | 0 | 0 | 3 | 2 | 1 | 1 | 1 |
| AT-rich element | binding site of AT-rich DNA binding protein (ATBP-1) | | 1 | 0 | 0 | 0 | 0 | 0 | 0 | 0 |
| ATCT-motif | part of a conserved DNA module involved in light responsiveness | | 0 | 1 | 1 | 1 | 1 | 0 | 0 | 1 |
| Box I | light responsive element | | 5 | 0 | 0 | 0 | 0 | 1 | 1 | 1 |
| Box II | part of a light responsive element | | 0 | 1 | 1 | 0 | 0 | 0 | 0 | 0 |
| Box III | protein binding site | | 0 | 0 | 0 | 0 | 0 | 0 | 0 | 1 |
| Box 4 | part of a conserved DNA module involved in light responsiveness | | 1 | 10 | 10 | 1 | 2 | 0 | 0 | 0 |
| CAT-box | cis-acting regulatory element related to meristem expression | | 0 | 0 | 0 | 0 | 1 | 1 | 1 | 0 |
| CAAT-box | common cis-acting element in promoter and enhancer regions | | 22 | 21 | 21 | 29 | 28 | 10 | 10 | 30 |
| CATT-motif | part of light responsive element | | 1 | 0 | 0 | 1 | 0 | 0 | 0 | 2 |
| CCAAT-box | MYBHv1 binding site | | 1 | 1 | 1 | 0 | 1 | 0 | 0 | 1 |
| CCGTCC-box | cis-acting regulatory element related to meristem specific activation | | 0 | 0 | 0 | 0 | 0 | 0 | 0 | 1 |
| CGTCA-motif | cis-acting regulatory element involved in the MeJA-responsiveness | | 2 | 1 | 1 | 1 | 1 | 0 | 0 | 0 |
| [EIRE](http://bioinformatics.psb.ugent.be/webtools/plantcare/cgi-bin/show_site_info.htpl?QWhere=ID_of_Site like 'NT~EIRE'&StartAt=0&NbRecs=10) | elicitor-responsive element | | 1 | 0 | 0 | 0 | 0 | 0 | 0 | 0 |
| ERE | ethylene-responsive element | | 4 | 0 | 0 | 0 | 0 | 0 | 0 | 1 |
| G-Box | cis-acting regulatory element involved in light responsiveness | | 2 | 7 | 7 | 4 | 1 | 0 | 0 | 0 |
| [GA-motif](http://bioinformatics.psb.ugent.be/webtools/plantcare/cgi-bin/show_site_info.htpl?QWhere=ID_of_Site like 'HA~GA-motif'&StartAt=0&NbRecs=10) | part of a light responsive element | | 1 | 0 | 0 | 1 | 0 | 0 | 0 | 0 |
| [GAG-motif](http://bioinformatics.psb.ugent.be/webtools/plantcare/cgi-bin/show_site_info.htpl?QWhere=ID_of_Site like 'SO~GAG-motif'&StartAt=0&NbRecs=10) | part of a light responsive element | | 2 | 2 | 2 | 0 | 1 | 1 | 1 | 0 |
| [GATA-motif](http://bioinformatics.psb.ugent.be/webtools/plantcare/cgi-bin/show_site_info.htpl?QWhere=ID_of_Site like 'AT~GATA-motif'&StartAt=0&NbRecs=10) | part of a light responsive element | | 1 | 1 | 1 | 0 | 2 | 1 | 1 | 0 |
| Gap-box | part of a light responsive element | | 0 | 0 | 0 | 0 | 1 | 0 | 0 | 0 |
| GARE-motif | gibberellin-responsive element | | 1 | 0 | 0 | 1 | 2 | 0 | 0 | 0 |
| GCN4_motif | cis-regulatory element involved in endosperm expression | | 0 | 1 | 1 | 1 | 1 | 0 | 0 | 2 |
| GT1-motif | light responsive element | | 0 | 1 | 1 | 1 | 3 | 0 | 0 | 0 |
| HD-Zip 3 | protein binding site | | 0 | 0 | 0 | 0 | 1 | 0 | 0 | 1 |
| [HSE](http://bioinformatics.psb.ugent.be/webtools/plantcare/cgi-bin/show_site_info.htpl?QWhere=ID_of_Site like 'BO~HSE'&StartAt=0&NbRecs=10) | cis-acting element involved in heat stress responsiveness | | 0 | 4 | 4 | 3 | 0 | 0 | 0 | 0 |
| I-Box | part of a light responsive element | | 2 | 2 | 2 | 2 | 2 | 1 | 1 | 0 |
| [LTR](http://bioinformatics.psb.ugent.be/webtools/plantcare/cgi-bin/show_site_info.htpl?QWhere=ID_of_Site like 'HV~LTR'&StartAt=0&NbRecs=10) | cis-acting element involved in low-temperature responsiveness | | 0 | 0 | 0 | 2 | 1 | 1 | 1 | 0 |
| MBS | MYB binding site involved in drought-inducibility | | 1 | 1 | 1 | 0 | 0 | 0 | 0 | 2 |
| [MBSI](http://bioinformatics.psb.ugent.be/webtools/plantcare/cgi-bin/show_site_info.htpl?QWhere=ID_of_Site like 'PH~MBSI'&StartAt=0&NbRecs=10) | MYB binding site involved in flavonoid biosynthetic genes regulation | | 0 | 0 | 0 | 1 | 0 | 0 | 0 | 0 |
| MRE | MYB binding site involved in light responsiveness | | 0 | 0 | 0 | 0 | 0 | 0 | 0 | 1 |
| MSA-like | cis-acting element involved in cell cycle regulation | | 1 | 0 | 0 | 0 | 0 | 0 | 0 | 0 |
| O2-site | cis-acting regulatory element involved in zein metabolism regulation | | 0 | 1 | 1 | 0 | 0 | 0 | 0 | 0 |
| P-box | gibberellin-responsive element | | 2 | 0 | 0 | 0 | 0 | 0 | 0 | 1 |
| RY-element | cis-acting regulatory element involved in seed-specific regulation | | 0 | 1 | 1 | 0 | 0 | 0 | 0 | 0 |
| Skn-1_motif | cis-acting regulatory element required for endosperm expression | | 1 | 1 | 1 | 5 | 0 | 1 | 1 | 1 |
| Sp1 | light responsive element | | 2 | 0 | 0 | 1 | 1 | 1 | 1 | 1 |
| TATA-box | core promoter element around -30 of transcription start | | 43 | 98 | 98 | 68 | 54 | 11 | 11 | 33 |
| TATC-box | cis-acting element involved in gibberellin-responsiveness | | 2 | 0 | 0 | 0 | 1 | 0 | 0 | 0 |
| TATCCAT/C-motif | NA | | 0 | 1 | 1 | 0 | 0 | 0 | 0 | 1 |
| [TC-rich repeats](http://bioinformatics.psb.ugent.be/webtools/plantcare/cgi-bin/show_site_info.htpl?QWhere=ID_of_Site like 'NT~TC-rich repeats'&StartAt=0&NbRecs=10) | cis-acting element involved in defense and stress responsiveness | | 0 | 3 | 3 | 1 | 0 | 0 | 0 | 0 |
| TCA-element | cis-acting element involved in salicylic acid responsiveness | | 0 | 1 | 1 | 0 | 1 | 1 | 1 | 0 |
| [TCCC-motif](http://bioinformatics.psb.ugent.be/webtools/plantcare/cgi-bin/show_site_info.htpl?QWhere=ID_of_Site like 'SO~TCCC-motif'&StartAt=0&NbRecs=10) | part of a light responsive element | | 0 | 0 | 0 | 0 | 1 | 0 | 0 | 0 |
| [TCCACCT-motif](http://bioinformatics.psb.ugent.be/webtools/plantcare/cgi-bin/show_site_info.htpl?QWhere=ID_of_Site like 'PH~TCCACCT-motif'&StartAt=0&NbRecs=10) | NA | | 0 | 1 | 1 | 0 | 0 | 0 | 0 | 0 |
| TCT-motif | part of a light responsive element | | 0 | 1 | 1 | 0 | 1 | 0 | 0 | 0 |
| TGA-element | auxin-responsive element | | 1 | 2 | 2 | 0 | 0 | 0 | 0 | 1 |
| TGACG-motif | cis-acting regulatory element involved in the MeJA-responsiveness | | 0 | 1 | 1 | 1 | 1 | 0 | 0 | 0 |
| [TGG-motif](http://bioinformatics.psb.ugent.be/webtools/plantcare/cgi-bin/show_site_info.htpl?QWhere=ID_of_Site like 'GH~TGG-motif'&StartAt=0&NbRecs=10) | part of a light responsive element | | 0 | 1 | 1 | 0 | 0 | 0 | 0 | 0 |
| Unnamed__1 | NA | | 3 | 2 | 2 | 0 | 1 | 0 | 0 | 0 |
| Unnamed__2 | single-strand DNA-binding proteins site (ssDBP-1 and -2) | | 0 | 0 | 0 | 0 | 1 | 0 | 0 | 0 |
| Unnamed__3 | NA | | 3 | 1 | 1 | 0 | 1 | 0 | 0 | 0 |
| Unnamed__4 | NA | | 9 | 1 | 1 | 3 | 8 | 3 | 3 | 2 |
| Unnamed__5 | NA | | 1 | 1 | 1 | 0 | 0 | 0 | 0 | 0 |
| Unnamed__6 | NA | | 0 | 1 | 1 | 0 | 0 | 0 | 0 | 0 |
| [Unnamed__8](http://bioinformatics.psb.ugent.be/webtools/plantcare/cgi-bin/show_site_info.htpl?QWhere=ID_of_Site like 'GM~Unnamed__8'&StartAt=0&NbRecs=10) | NA | | 0 | 1 | 1 | 0 | 0 | 0 | 0 | 0 |
| [Unnamed__13](http://bioinformatics.psb.ugent.be/webtools/plantcare/cgi-bin/show_site_info.htpl?QWhere=ID_of_Site like 'ZM~Unnamed__13'&StartAt=0&NbRecs=10) | NA | | 0 | 0 | 0 | 1 | 0 | 0 | 0 | 0 |
| WUN-motif | wound-responsive element | | 0 | 0 | 0 | 1 | 0 | 0 | 0 | 1 |
| box S | NA | | 0 | 0 | 0 | 0 | 0 | 0 | 0 | 1 |
| chs-CMA1a | part of a light responsive element | | 1 | 0 | 0 | 0 | 0 | 0 | 0 | 0 |
| chs-CMA2a | part of a light responsive element | | 0 | 0 | 0 | 0 | 0 | 0 | 0 | 1 |
| circadian | cis-acting regulatory element involved in circadian control | | 1 | 2 | 2 | 2 | 2 | 0 | 0 | 4 |
| rbcS-CMA7a | part of a light responsive element | | 1 | 0 | 0 | 0 | 0 | 0 | 0 | 0 |
| [dOCT](http://bioinformatics.psb.ugent.be/webtools/plantcare/cgi-bin/show_site_info.htpl?QWhere=ID_of_Site like 'AT~dOCT'&StartAt=0&NbRecs=10) | cis-acting regulatory element related to meristem specific activation | | 0 | 1 | 1 | 0 | 0 | 0 | 0 | 0 |

**Supplementary Table 3.** Expression analysis of RNA-seq data from representatives HSD1 and HSD5 genes in different tissues

| **Species/Gene Name** | **NCBI BioProject ID** | **NCBI Accession No.** | **Important Note** |
| --- | --- | --- | --- |
| A. hypochondriacus HSD1 | PRJNA290113 | SRR5346226 (PacBio),  SRR5345531 (Hi-C),  SRR5518360 (GBS), | Bulk data downloads, BLAST analysis, and JBrowse viewing of the final proximity-guided assembly are available at Phytozome (https://phytozome.jgi.doe.gov/Ahypochondriacus_er) and CoGe (https://genomevolution.org/coge/; Genome ID 34733). |
| A. hypochondriacus HSD5 | PRJNA290113 | SRR5346226 (PacBio),  SRR5345531 (Hi-C), SRR5518360 (GBS), | Bulk data downloads, BLAST analysis, and JBrowse viewing of the final proximity-guided assembly are available at Phytozome (https://phytozome.jgi.doe.gov/Ahypochondriacus_er) and CoGe (https://genomevolution.org/coge/; Genome ID 34733). |
| G. max HSD1 | [PRJNA19861](https://www.ncbi.nlm.nih.gov/bioproject/PRJNA19861),  [PRJNA48389](https://www.ncbi.nlm.nih.gov/bioproject/PRJNA48389) | PRJNA48389,  GCA_002905335.1,  GCA_002907465.1, | Assemblies and annotations are also available for download and browsing at both Phytozome (https://phytozome.jgi.doe.gov) and SoyBase (https://soybase.org/data/public/Glycine_max/). |
| G. max HSD5a | [PRJNA19861](https://www.ncbi.nlm.nih.gov/bioproject/PRJNA19861),  [PRJNA48389](https://www.ncbi.nlm.nih.gov/bioproject/PRJNA48389) | PRJNA48389,  GCA_002905335.1,  GCA_002907465.1, | Assemblies and annotations are also available for download and browsing at both Phytozome (https://phytozome.jgi.doe.gov) and SoyBase (https://soybase.org/data/public/Glycine_max/). |
| G. max HSD5b | [PRJNA19861](https://www.ncbi.nlm.nih.gov/bioproject/PRJNA19861),  [PRJNA48389](https://www.ncbi.nlm.nih.gov/bioproject/PRJNA48389) | PRJNA48389,  GCA_002905335.1,  GCA_002907465.1, | Assemblies and annotations are also available for download and browsing at both Phytozome (https://phytozome.jgi.doe.gov) and SoyBase (https://soybase.org/data/public/Glycine_max/). |

**Reference Publication(s)**

- Lightfoot, D. J., Jarvis, D. E., Ramaraj, T., Lee, R., Jellen, E. N., & Maughan, P. J. (2017). Single-molecule sequencing and Hi-C-based proximity-guided assembly of amaranth (Amaranthus hypochondriacus) chromosomes provide insights into genome evolution. *BMC Biology*, *15*(1). <https://doi.org/10.1186/s12915-017-0412-4>
- Clouse, J. W., Adhikary, D., Page, J. T., Ramaraj, T., Deyholos, M. K., Udall, J. A., … Maughan, P. J. (2016). The Amaranth Genome: Genome, Transcriptome, and Physical Map Assembly. *The Plant Genome*, *9*(1). <https://doi.org/10.3835/plantgenome2015.07.0062>
- Valliyodan, B., Cannon, S. B., Bayer, P. E., Shu, S., Brown, A. V., Ren, L., … Nguyen, H. T. (2019). Construction and comparison of three reference‐quality genome assemblies for soybean. *The Plant Journal*, *100*(5), 1066–1082. <https://doi.org/10.1111/tpj.14500>
- Schmutz, J., Cannon, S. B., Schlueter, J., Ma, J., Mitros, T., Nelson, W., … Jackson, S. A. (2010). Genome sequence of the palaeopolyploid soybean. *Nature*, *463*(7278), 178–183. <https://doi.org/10.1038/nature08670>
